# A semi-mechanistic exposure–response model to assess the effects of verinurad, a potent URAT1 inhibitor, on serum and urine uric acid in patients with hyperuricemia-associated diseases

**DOI:** 10.1007/s10928-021-09747-y

**Published:** 2021-03-17

**Authors:** Jacob Leander, Mikael Sunnåker, Dinko Rekić, Sergey Aksenov, Ulf G. Eriksson, Susanne Johansson, Joanna Parkinson

**Affiliations:** 1 Clinical Pharmacology and Quantitative Pharmacology, Clinical Pharmacology and Safety Sciences, R&D, AstraZeneca, Gothenburg, Sweden; 2 Clinical Pharmacology and Quantitative Pharmacology, Clinical Pharmacology and Safety Sciences, R&D, AstraZeneca, Waltham, MA USA

**Keywords:** Uric acid, PKPD, Hyperuricemia, Semi-mechanistic modeling, URAT1, Mixed effects modeling, Verinurad, Febuxostat, Allopurinol

## Abstract

Verinurad, a uric acid transporter 1 (URAT1) inhibitor, lowers serum uric acid by promoting its urinary excretion. Co-administration with a xanthine oxidase inhibitor (XOI) to simultaneously reduce uric acid production rate reduces the potential for renal tubular precipitation of uric acid, which can lead to acute kidney injury. The combination is currently in development for chronic kidney disease and heart failure. The aim of this work was to apply and extend a previously developed semi-mechanistic exposure–response model for uric acid kinetics to include between-subject variability to verinurad and its combinations with XOIs, and to provide predictions to support future treatment strategies. The model was developed using data from 12 clinical studies from a total of 434 individuals, including healthy volunteers, patients with hyperuricemia, and renally impaired subjects. The model described the data well, taking into account the impact of various patient characteristics such as renal function, baseline fractional excretion of uric acid, and race. The potencies (EC50s) of verinurad (reducing uric acid reuptake), febuxostat (reducing uric acid production), and oxypurinol (reducing uric acid production) were: 29, 128, and 13,030 ng/mL, respectively. For verinurad, symptomatic hyperuricemic (gout) subjects showed a higher EC50 compared with healthy volunteers (37 ng/mL versus 29 ng/mL); while no significant difference was found for asymptomatic hyperuricemic patients. Simulations based on the uric acid model were performed to assess dose–response of verinurad in combination with XOI, and to investigate the impact of covariates. The simulations demonstrated application of the model to support dose selection for verinurad.

**Supplementary Information:**

The online version contains supplementary material available at 10.1007/s10928-021-09747-y.

## Introduction

Hyperuricemia, defined as elevated serum uric acid (sUA) levels, has been linked to an increased risk of kidney disease, hypertension, coronary heart disease, cardiovascular mortality, and diabetic retinopathy [1–7]. These discoveries raised the question of whether sUA lowering therapy would improve patient outcomes; at present there is no clear and consistent evidence that sUA lowering translates into cardiovascular and renal benefits, with some trials reporting improvement in patients, while others showing no benefit or even worsening [8–10]. Verinurad, which lowers sUA levels, is currently in development in combination with xanthine oxidase inhibitor (XOI) for chronic kidney disease (CKD) and heart failure (HF). The combination has shown promising results reducing albuminuria, a surrogate marker of early kidney disease [11].

The production of uric acid occurs via purine degradation and is catalyzed by xanthine oxidase (XO), while excretion occurs mainly by the kidneys [12]. Most uric acid is reabsorbed in the proximal tubules by uric acid transporter 1 (URAT1) [13], as evidenced by approximately 5- to tenfold higher fractional excretion (i.e. 50–80%) in individuals with inactivating mutations in the URAT1 gene than normal individuals [14]. In addition to URAT1, solute carrier family 2 facilitated glucose transporter member 9 (GLUT9) and organic iron transporter 4 (OAT4) reabsorb uric acid from the renal tubular lumen back to the systemic circulation.

Hyperuricemia may occur due to either overproduction and/or underexcretion of uric acid. Current options for treating hyperuricemia include inhibitors of uric acid production, such as the XOIs febuxostat and allopurinol, and older uricosurics promoting uric acid excretion (including via URAT1 inhibition) such as benzbromarone, probenecid, sulfinpyrazone, and the selective URAT1 inhibitor lesinurad. Lesinurad 200 mg was approved in combination with allopurinol or febuxostat by the United States Food and Drug Administration (FDA) for treatment of hyperuricemia associated with gout [15].

Verinurad is a novel, selective uric acid reabsorption inhibitor that inhibits URAT1 in the renal tubular lumen, leading to increased urinary excretion of uric acid and decreased sUA levels, and has increased potency for URAT1 compared with lesinurad [16]. Verinurad in combination with a XOI (either febuxostat or allopurinol) has shown effective reduction of sUA levels in patients with hyperuricemia associated with gout [17–19]. Importantly, the combination of verinurad and XOI was more effective in reducing sUA levels than either alone [20, 21]. In addition to intensive serum urate lowering, verinurad and XOI treatment was shown to be safe, with minimal effect on urinary uric acid (uUA) levels in phase 1 and 2 studies in adults with gout [18, 19, 21]. In contrast to verinurad, increased incidence of renal adverse events have been observed with the highest dose (400 mg) of lesinurad in combination with a XOI, possibly due to microcrystallization of uUA in renal tubules [22].

The effect of urate-lowering therapies, such as XOIs and URAT1 inhibitors, may be affected by underlying pathophysiological situations such as impaired renal function. A semi-mechanistic exposure–response model for uric acid dynamics has previously been developed to assess the combined effect of the URAT1 inhibitor lesinurad and a XOI to predict mean response [23]. The aim of the present work was to apply the uric acid model structure to data collected during the verinurad clinical development program and estimate uric acid model parameters, as well as drug effects, on uric acid dynamics. In addition, the previously developed mean response model was extended to a mixed effects model to include between-subject variability on uric acid parameters. A variety of simulations were conducted to support use of the model for dose selection for future studies with verinurad.

## Methods

### Clinical studies

All available clinical data from studies where modified release (MR4) or extended release (ER8) verinurad formulations were used in the present analysis, comprising 12 clinical studies of verinurad and including single ascending dose (SAD), multiple ascending dose (MAD), drug-drug interaction (DDI), and several phase 2a studies in several patient populations. For a detailed overview of the clinical studies, see Table 1. Sampling schedules for each study are listed in Supplementary materials 1–3. In total, 434 individuals were incorporated in the integrated analysis.

**Table 1:** Overview of clinical data used for analysis

Study	Description	Subjects	Population	Verinurad doses (mg)	XOI doses (mg)	Reference
RDEA3170-104	Single and Multiple Dose Study in Japanese Subjects	48	Healthy volunteers	2.5, 5, 10, 15^a^	–	NCT01872832; [41]
RDEA3170-105	Verinurad and Febuxostat Drug Interaction Study	23	Healthy volunteers	2.5, 10^a^	40 mg febuxostat	NCT01883167; [20]
RDEA3170-107	Verinurad and Allopurinol Combination Study in Gout Subjects	12	Symptomatic hyperuricemic	10^a^	300 mg allopurinol	NCT02279641; [21]
RDEA3170-108	PK Renal Impairment Study	31	Mild, moderate, and severe renal impairment, normal renal function	15^a^	–	NCT02219516; [38]
RDEA3170-110	Bioavailability Study	15	Healthy volunteers	10^a^	–	NCT02336594
RDEA3170-112	Single and Multiple Dose Study	40	Healthy volunteers	4.5, 6, 12^b^	–	NCT02608710
RDEA3170-204	Verinurad and Febuxostat Combination Study	64	Symptomatic hyperuricemic	2.5, 5, 10, 15, 20^a^	40, 80 mg febuxostat	NCT02246673; [18]
RDEA3170-205	Verinurad and Febuxostat Combination Study	72	Symptomatic hyperuricemic	2.5, 5, 10, 15^a^	10, 20, 40 mg febuxostat	NCT02317861; [19]
RDEA3170-206	Phase 2a Verinurad and Allopurinol Combination Study in Gout Subjects	41	Symptomatic hyperuricemic	2.5, 7.5, 15, 20^a^	300 (qd and bid), 600 mg allopurinol	NCT02498652; [17]
D5495C00001	PD DDI with verinurad, febuxostat, and dapagliflozin	36	Asymptomatic hyperuricemic	9^b^	80 mg febuxostat	NCT03118739
D5495C00006*	Multiple dose study in Asians/Chinese	18	Healthy volunteers	12, 24^b^	300 mg allopurinol	NCT03836599
D5495C00007	Ph2a, Verinurad and Febuxostat in Patients With Albuminuria	60	T2DM, asymptomatic hyperuricemic, with albuminuria	9^b^	80 mg febuxostat	NCT03118739

*XOI* xanthine oxidase inhibitor

^*^Used for model validation

^a^Modified release (MR4) formulation

^b^Extended release (ER8) formulation

### Pharmacokinetic models

Population pharmacokinetic (popPK) models were developed separately for verinurad (modified release [MR4] and extended release formulations [ER8]) and oxypurinol (active metabolite of allopurinol), in order to better capture the complex absorption of each formulation and obtain individual PK parameter estimates for each subject. Details of the popPK models can be found in the Supplementary Material. For febuxostat, a previously published popPK model was used with no modifications [24].

### Pharmacodynamic model of uric acid handling

**Fig. 1:**
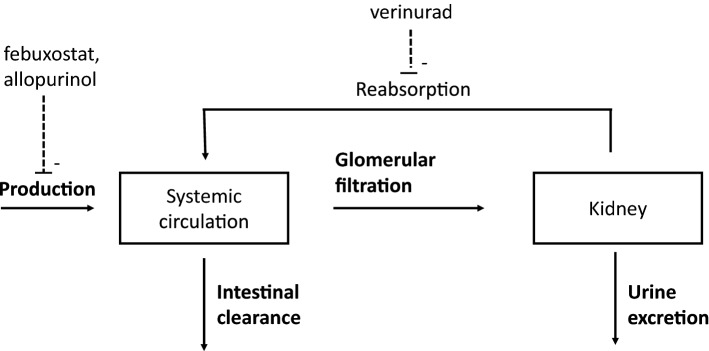
Schematic view of the verinurad uric acid model with the key processes and drug actions shown. Febuxostat and allopurinol inhibits the production of uric acid while verinurad inhibits the reabsorption (dashed lines)

To model the uric acid in circulation and urine, a semi-mechanistic model of uric acid disposition was used as a structural model [23], and is reproduced here for the reader’s convenience. An illustrative graphic of the model is shown in Fig. 1.

The key kinetic processes that constitute mass balance of uric acid in the body are production of uric acid, intestinal clearance, and renal clearance. The rate of change (mg/h) of the amount of uric acid in the systemic compartment A1 (mg) is given by the differential equation:

1 $$\frac{{dA_{1} }}{dt} = R_{P} - R_{I} - R_{R} ,$$

where $R_{P}$ is the production rate, $R_{I}$ is intestinal elimination rate and $R_{R}$ is the renal excretion rate. The rate of intestinal elimination of uric acid (mg/h) is described by a first order kinetic process with respect to concentration of uric acid in the systemic compartment $C_{UA}$ (mg/L) according to:

2 $$R_{I} = CL_{{\text{I}}} *C_{UA}$$

where $CL_{{\text{I}}}$ is the intestinal clearance of uric acid (L/h). Concentration of uric acid in the systemic compartment is given by:

3 $$C_{UA} = \frac{{A_{1} }}{{V_{1} }}$$

where $V_{1}$ is the volume of distribution (L).

Inhibition of the production rate by febuxostat or oxypurinol is described by a saturable function of drug concentration in plasma with parameters $E_{max}$ and $EC_{50}$ specific to each XOI drug. The value 1 − $E_{max}$ corresponds to the maximum possible inhibition of the production rate of uric acid achieved with a very large dose of allopurinol or febuxostat. The value of *EC*_*50*_ is the plasma concentration of the drug that results in half-maximal inhibition of the production rate. The rate $k_{{{\text{in}}}}$ (mg/h) is production rate of uric acid in the absence of drug treatment.

4 $$R_{P} = k_{{{\text{in}}}} \left( {1 - \frac{{E_{max} C_{x} }}{{EC_{50} + C_{X} }}} \right)$$

where $C_{x}$ corresponds to the plasma concentration of either febuxostat or oxypurinol.

Key kinetic processes that constitute renal handling of uric acid are glomerular filtration of uric acid and renal excretion of uric acid. Uric acid is freely filtered into the proximal tubule and is reabsorbed via urate transporters back into the systemic circulation [25]. The input rate of filtration (also known as the filtered load) of uric acid into the kidney from the circulation is denoted $FL_{in}$ (mg/h), which is modelled to be equal to the concentration of uric acid in the systemic compartment multiplied by the glomerular filtration flow rate (GFR). In this work, the GFR is approximated using the CKD-EPI formula [26]. In the following equations, estimated GFR (eGFR) is given in L/h (1 ml/min/1.73 m^2^ = 0.06 L/h) to have consistent units. Hence, the filtration rate of uric acid into the kidney is given by the relationship:

5 $$FL_{in} = eGFR{*}C_{UA}$$

The rate of renal excretion of uric acid is calculated as the product of the input filtration rate and the fractional excretion coefficient (FEUA).

6 $$R_{R} = FL_{in} {*}FEUA = eGFR{*}C_{UA} *FEUA$$

Verinurad increases the excretion rate by inhibiting the reabsorption of uric acid back to the circulation. An Emax model is used to describe the stimulating drug action of the FEUA according to the relationship:

7 $$R_{R} = eGFR{*}C_{UA} *\left( {FEUA + \frac{{E_{max} C_{V} }}{{EC_{50} + C_{V} }}} \right)$$

where $C_{V}$ denotes the plasma concentration of verinurad. The value $FEUA$ + $E_{max}$ is the maximum possible fractional excretion of uric acid achieved with a very large dose of verinurad. The value of *EC*_*50*_ is the plasma concentration of verinurad that results in half-maximal inhibition of reabsorption, i.e. when the fractional excretion is $FEUA$ + E_max_/2. The renal excretion rate $R_{R}$ is the rate of accumulation of uric acid in the bladder and can be indirectly estimated by measuring accumulation of uric acid in final, collected urine.

The full system of differential equations of the model are the following, where $A_{1}$ is the amount of uric acid (in mg) in the systemic circulation and $A_{2}$ is the cumulative amount of uric acid (in mg) excreted in urine:

8 $$\begin{gathered} \frac{{dA_{1} }}{dt} = k_{{{\text{in}}}} \left( {1 - \frac{{E_{max} C_{X} }}{{EC_{50} + C_{X} }}} \right) - CL_{{\text{I}}} \frac{{A_{1} }}{{V_{1} }} - eGFR{*}\frac{{A_{1} }}{{V_{1} }}*\left( {FEUA + \frac{{E_{max} C_{V} }}{{EC_{50} + C_{V} }}} \right) \hfill \\ \frac{{dA_{2} }}{dt} = eGFR{*}\frac{{A_{1} }}{{V_{1} }}*\left( {FEUA + \frac{{E_{max} C_{V} }}{{EC_{50} + C_{V} }}} \right) \hfill \\ A_{1} \left( 0 \right) = \frac{{V_{1} k_{{{\text{in}}}} }}{{CL_{{\text{I}}} + eGFR{*}FEUA}} \hfill \\ A_{2} \left( 0 \right) = 0 \hfill \\ \end{gathered}$$

where the initial condition $A_{1} \left( 0 \right)$ is calculated from steady-state assumption (solving the equation $\frac{{dA_{1} }}{dt} = 0$), and the initial condition for the cumulative amount in urine, $A_{2} \left( 0 \right),$ is set to zero.

The baseline concentration of uric acid, assuming steady-state conditions without the presence of drug, is given by the amount in the systemic circulation at baseline, $A_{1} \left( 0 \right)$, divided by the volume of distribution $V_{1}$:

9 $$(C_{UA} )_{SS} = \frac{{A_{1} \left( 0 \right)}}{{V_{1} }} = { }\frac{{k_{{{\text{in}}}} }}{{CL_{{\text{I}}} + eGFR{*}FEUA}}$$

which has the unit mg/L.

In Aksenov et al. the semi-mechanistic uric acid model was developed as a mean response model, with the aim of describing the mean response in the population [23]. In this work, we consider the extension to a mixed-effects model, with the ability to describe both mean response and variability in the population. In a mixed-effects model, parameters are modeled with statistical distributions to describe variability between individuals. We specified lognormal distributions for model parameters $\theta_{i}$ as follows:

10 $$\theta_{i} = \theta_{pop} {\text{ exp}}\left( \eta \right)$$

where $\theta_{pop} { }$ is the typical value of the parameter in the population (median of the population distribution), and $\eta$ is the random effect, normally distributed with mean zero and standard deviation ω.

Residual error model for uric acid in serum and urine had additive and proportional variance components:

11 $${\varvec{y}} = {\varvec{f}} + \sqrt {{\varvec{a}}^{2} + \left( {{\varvec{b}} \times {\varvec{f}}} \right)^{2} } \times {\varvec{\varepsilon}}$$

where *y* is the observed uric acid in serum or urine, *f* is the model prediction, *a* is the standard deviation for the additive error component, *b* is the standard deviation for the proportional error component and ε is distributed independently and normally about mean 0 with variance 1. The residual error model for concentration of uric acid in serum was an additive model (i.e. *b* = 0); for uric acid in urine, a combined additive and proportional model was used.

### Development of the integrated uric acid model

The semi-mechanistic model was developed in following steps: first, three popPK models were developed for verinurad, febuxostat, and oxypurinol. As a second step, the individual PK time-profiles (using the empirical Bayes estimates) of the respective drug exposures were used to drive the PD uric acid model.

Discrimination between models such as various combinations of covariates or parameter variability or residual error models, was done by inspection of graphical diagnostics, precision of parameter estimates, and changes in the objective function value provided by NONMEM [27]. Inclusion of correlation between random effects (OMEGA block) was guided by correlation between empirical Bayes Estimates. The adequacy of the models was evaluated using graphical analysis of goodness-of-fit plots and visual predictive checks [28].

Study D5495C00006 was used as a validation study in the current assessment. The reason for this was that in this study, the design included a 7-day allopurinol run-in period and the first urinary samples were collected already after patients were treated with allopurinol monotherapy for 7 days. Therefore, a true baseline of uUA measurements, collected without any treatment, were not available. These measurements are required for accurate estimation of the baseline characteristics and consequently the magnitude of treatment effect. Therefore, the decision was made to use this study as a validation, instead of a part of the modeling dataset. The integrated uric acid model was used to produce visual predictive check plots for study D5495C00006, to assess whether the model can adequately predict results from an external study and thus validating its predictive value.

### Model simulations

Two types of simulations were applied: simulation with variability and simulation with uncertainty. Simulations with variability were performed using the estimated parameter values including patient variability. To simulate the individual response, the covariance matrix of the random effects (OMEGA matrix in NONMEM) was sampled for 500 individuals and the 5th, 50th, and 95th quantiles were calculated. For the simulation with uncertainty, a set of variable combinations were simulated (n = 500) using mean parameter values and the covariance matrix of model parameters. Next, a model prediction (e.g. sUA reduction or UA renal excretion rate) was derived for each variable combination; this resulted in 500 simulated outcomes that were then used to calculate a median prediction with 5% and 95% quintiles of the distribution. The mean covariate values were used in the simulations; details of specific covariate values used in each simulation can be found in the sections below.

### Temporal uric acid response to verinurad in combination with febuxostat

Simulations were performed assuming mean baseline characteristics representative of the CKD/HF patient population: eGFR = 60 mL/min/1.73 m^2^ and asymptomatic hyperuricemia (which resulted in baseline sUA of approximately 8.5 mg/dL and baseline FEUA = 7.7%). A 12 mg ER8 verinurad dose with or without an 80 mg febuxostat dose was used in the simulations.

### Dose–response for verinurad in combination with XOI in the CKD/HF population

Simulations were performed assuming mean baseline characteristics representative of the CKD/HF patient population as used for the temporal uric acid response to verinurad in combination with febuxostat. A dose range between 0 and 15 mg for the verinurad ER8 formulation, in combination with either 80 mg febuxostat or 300 mg allopurinol was simulated for generating dose–response curves.

### Assessment of the impact of covariates on the PK/PD of verinurad

For the assessment of covariates, each covariate was compared with a typical patient using the following typical values: eGFR of 60 mL/min/1.73 m^2^, body weight of 80 kg, and non-Asian race. The following range of covariates were explored: Asian versus non-Asian; eGFR 45, 60, and 90 mL/min/1.73 m^2^; body weight 50, 80, and 100 kg. A 12 mg ER8 verinurad dose combined with 80 mg febuxostat was used in the simulations.

### Selecting the dose of XOI for combination with verinurad

Simulations were performed assuming the following baseline characteristics: eGFR 60 mL/min/1.73 m^2^, asymptomatic hyperuricemia (which resulted in a baseline sUA of approximately 8.5 mg/dL and baseline FEUA = 7.7%), and non-Asian race. A 12 mg ER8 verinurad dose combined with the following febuxostat doses were used in the simulations: 0, 20, 40, 60, and 80 mg.

### Software

The popPK models and the uric acid model were estimated using NONMEM 7.3.0 [27]. For the popPK models the first order conditional estimation (FOCE) method was used, while the first order conditional estimation with interaction (FOCEI) was used for the uric acid model. R, version 3.5.1 [29] was used for the exploratory analyses. The R package nonmem2R, version 0.2.1 was used to produce visual predictive checks and goodness-of-fits plots [30]. The R package mrgsolve, version 0.8.12 [31] was used to perform the model simulations.

## Results

### Subject characteristics

A summary of the demographics and baseline characteristics of the subjects included in the analysis can be found in Table 2. The mean sUA and eGFR values were 7.6 mg/dL and 97 mL/min/1.73 m^2^, respectively. Subjects included in the analysis were mostly men (nearly 97% of the overall population) and 43% and 22% of patients had symptomatic and asymptomatic hyperuricemia, respectively. Overall, there was a wide spread in baseline sUA, with a range between 4 and 13.3 mg/dL.

**Table 2:** Summary of baseline characteristics of patients included in the analysis

Study	n	Age (years) median (min, max)	Body weight (kg) median (min, max)	eGFR (ml/min/1.73 m^2^) median (min, max)	sUA (mg/dL) median (min, max)	Sex (males) n (%)	Race: Caucasian n (%)	Race: Black n (%)	Race: Asian n (%)	Race: other n (%)
D5495C00001	36	41 (20, 63)	85.7 (57.7, 121.3)	91.4 (52.8, 128)	7.1 (5.8, 9.6)	35 (97.2)	14 (38.9)	17 (47.2)	2 (5.6)	3 (8.3)
D5495C00006	21	38 (27, 48)	72.25 (58.75, 90.75)	103 (70.4, 119.1)	6 (4.2, 10.7)	19 (90.5)	0 (0)	0 (0)	21 (100)	0 (0)
D5495C00007	32	62 (43, 79)	90.4 (52.6, 136.3)	55.9 (31.6, 109.9)	7.5 (6, 13.3)	22 (68.8)	22 (68.8)	6 (18.8)	3 (9.4)	1 (3.1)
RDEA3170-104	48	31.5 (21, 53)	70.4 (54.9, 101.7)	113.4 (78, 138.3)	5.7 (4, 8.1)	48 (100)	5 (10.4)	3 (6.2)	40 (83.3)	0 (0)
RDEA3170-105	23	35 (21, 48)	87.9 (65.8, 115.9)	98.3 (75.7, 117.7)	6.4 (5, 9.1)	23 (100)	15 (65.2)	7 (30.4)	0 (0)	1 (4.3)
RDEA3170-107	12	51.5 (29, 69)	99.7 (80.9, 126.3)	84.3 (54.1, 102.9)	8.6 (7.1, 9.6)	12 (100)	7 (58.3)	4 (33.3)	0 (0)	1 (8.3)
RDEA3170-108	31	61 (35, 81)	82.6 (59, 122)	74 (12.3, 106.1)	6.7 (4.1, 12.4)	31 (100)	23 (74.2)	7 (22.6)	0 (0)	1 (3.2)
RDEA3170-110	15	44 (25, 62)	90.9 (65.4, 129.4)	99.2 (74.9, 134.3)	5.3 (4.4, 6.5)	15 (100)	11 (73.3)	2 (13.3)	0 (0)	2 (13.3)
RDEA3170-112	40	35.5 (24, 56)	86.1 (59.2, 126.8)	99.6 (78.1, 131.6)	5.7 (4.2, 7.4)	40 (100)	22 (55)	17 (42.5)	1 (2.5)	0 (0)
RDEA3170-204	64	48 (29, 71)	98.65 (63.8, 138.7)	89.7 (57.4, 121.1)	8.8 (5.5, 12.3)	64 (100)	43 (67.2)	7 (10.9)	14 (21.9)	0 (0)
RDEA3170-205	72	46 (21, 67)	75.45 (58.3, 115.6)	105.6 (76.8, 133.2)	8.6 (7.5, 12.7)	72 (100)	0 (0)	0 (0)	72 (100)	0 (0)
RDEA3170-206	40	48 (28, 74)	95.5 (63.1, 147.6)	89.9 (55.1, 125.6)	9 (6.7, 10.7)	39 (97.5)	30 (75)	6 (15)	4 (10)	0 (0)
All subjects	434	45 (20, 81)	84 (52.6, 147.6)	97 (12.3, 138.3)	7.6 (4, 13.3)	420 (96.8)	192 (44.2)	76 (17.5)	157 (36.2)	9 (2.1)

*eGFR* estimated glomerular filtration rate, *sUA* serum uric acid

### Pharmacokinetics of verinurad, febuxostat, and oxypurinol

PopPK models developed for verinurad and oxypurinol (allopurinol active metabolite) fitted data well, as judged by goodness of fit plots and precision of parameter estimates.

Two separate PK models were developed for verinurad: one for modified release (MR4) and one for extended release (ER8) formulations, with different formulation principles (MR4 as a tablet and ER8 as a capsule) and bioavailability. Both models were two-compartmental disposition models with an absorption comprising of zero-order infusion into the dosing compartment and sequential first-order absorption. eGFR and body weight were found to be significant covariates on verinurad clearance.

For oxypurinol, a two-compartmental disposition model with first-order absorption was used. eGFR was found to be covariate on clearance, while body weight was used for central volume of distribution. As expected, verinurad was found to have an impact on oxypurinol exposure (concomitant intake of verinurad reduces oxypurinol exposure) [21]. Detailed results from popPK modeling for verinurad and oxypurinol, including final parameter estimates and goodness-of-fit plots, can be found in the Supplementary Material.

### Estimation of model parameters

The estimated parameters for the final model are listed in Table 3. The covariate effect on FEUA was modelled as:

12 $${\text{FEUA }} = {\text{ FEUA}}\_{\text{TYP * }}\left( {1 + {\text{HYPERFEUA}}} \right){ * }\left( {1 + {\text{ASIANFEUA}}} \right),$$

where FEUA_TYP corresponds to FEUA in healthy volunteers and HYPERFEUA and ASIANFEUA are the covariate estimates corresponding to hyperuricemic patients (either symptomatic or asymptomatic) and Asians, respectively.

**Table 3:** Estimated parameter values of the verinurad and XOI uric acid model

Parameter	Typical value	RSE %
${k}_{\mathrm{in}}$, (mg/h)	43.78	1.75
FEUA, healthy volunteers	0.0906	3.18
CL_I_ (L/h)	0.2272	12.8
V_1_, uric acid (L)	14.11	0.661
Verinurad EC50 (ng/mL)	29.40	4.73^a^
Febuxostat EC50 (ng/mL)	128.0	7.16^a^
Oxypurinol EC50 (ng/mL)	13,030	12^a^
Residual error, additive, serum	0.4107	0.158
Residual error, additive, urine	3.054	0.537
Residual error, proportional, urine	0.3806	0.371
Residual error, additive, serum study D5495C00006	0.9524	2.4
FEUA covariate, asymptomatic hyperuricemic	− 0.1501	32.2
FEUA covariate, symptomatic hyperuricemic (gout)	− 0.3787	6.06
FEUA covariate, Asians	− 0.2322	11.5
Verinurad EC50 covariate, symptomatic hyperuricemic (gout)	0.2677	33.5
${k}_{\text{in}}$, IIV (CV%)	27.76	3.34
FEUA, IIV, (CV%)	42.54	3.98
Verinurad EC50, IIV, (CV%)	57.00	3.05
Febuxostat EC50, IIV, (CV%)	89.96	3.40
Oxypurinol EC50, IIV, (CV%)	65.44	9.66
Off diagonal element production rate—FEUA, (cor)	0.761	4.20

The covariate effect on FEUA was modelled as: FEUA = FEUA_TYP * (1 + HYPERFEUA) * (1 + ASIANFEUA), where FEUA_TYP corresponds to FEUA in healthy volunteers and HYPERFEUA and ASIANFEUA are the covariate estimates corresponding to hyperuricemic (either symptomatic or asymptomatic) and Asians, respectively

The covariate effect on verinurad EC50 was modelled as: EC50 = EC50_TYP *(1 + HYPEREC50), where EC50_TYP corresponds to EC50 in healthy volunteers and HYPEREC50 corresponds to the covariate estimate for symptomatic hyperuricemic (gout) patients

*CL*_*I*_ intestinal clearance of uric acid, *FEUA* fractional excretion uric acid (here, reported as fraction), *HYPEREC50* verinurad EC50 covariate in symptomatic hyperuricemic patients, *IIV* interindividual variability, *k*_*in*_ uric acid production rate, *R*_*P*_ uric acid production rate, *RSE* relative standard error, *V*_*1*_ volume of distribution

^a^Estimated on log-scale and reported as CV%

The covariate effect on verinurad EC50 was modelled as:

13 $$EC50 = EC50\_TYP *\left( {1 + HYPEREC50} \right),$$

where EC50_TYP corresponds to EC50 in healthy volunteers and HYPEREC50 corresponds to the covariate estimate for symptomatic hyperuricemic (gout) patients. Parameters were estimated with adequate precision; relative standard errors (RSE) were less than 35%, with the majority of parameters having RSE below 30%. Despite some evidence of a misfit to uUA data and overprediction of the steady state sUA data, in general the goodness-of-fit and visual predictive check plots support the adequacy of the model for the intended purpose of supporting planning and decision making for future studies (Supplementary Material 4).

Uric acid baseline fractional excretion, FEUA, was found to be different between healthy volunteers and patients with hyperuricemia. Interestingly, baseline FEUA also differed between patients with symptomatic (gout) and asymptomatic hyperuricemia. The baseline value was higher in healthy volunteers (9.1%) compared with the symptomatic (5.6%) and asymptomatic (7.7%) hyperuricemic patients. Moreover, Asian subjects were found to have a slightly lower fractional excretion of uric acid compared with non-Asians (4.3% compared with 5.6% in symptomatic hyperuricemic patients). It should be noted that only patients from study D5495C00001 were used to estimate the separate FEUA value for asymptomatic hyperuricemic patients. Asymptomatic hyperuricemics were also included in study D5495C00007, however, no urine collection was performed in this study and therefore it was not possible to reliably estimate FEUA for those patients.

Emax models were able to adequately describe the effects of verinurad, febuxostat, and oxypurinol. It was not possible to reliably estimate the maximum effects (Emax) of each drug using current data. Therefore, those parameters were fixed; the maximum effects for febuxostat and oxypurinol were fixed to the same values as in Aksenov et al. 2018 (1 and 0.84 for febuxostat and oxypurinol Emax, respectively; this corresponds to the maximum inhibition of uric acid production) [23]. For verinurad, the Emax parameter corresponds to the maximum increase in fractional excretion of uric acid; this value was fixed to the 0.7, consistent with FEUA values reported in people with loss of function mutations in URAT1 [14, 32]. The potencies (EC50s) of verinurad, febuxostat, and oxypurinol were: 29.3, 128, and 13,030 ng/mL, respectively. It was explored whether potency was different between healthy volunteers and hyperuricemic patients. For verinurad, symptomatic hyperuricemic (gout) patients showed a higher EC50 compared with healthy volunteers (37.3 ng/mL versus 29.3 ng/mL); for the asymptomatic hyperuricemic patients (studies D5495C00001 and D5495C00007), no significant difference versus healthy volunteers could be established. For febuxostat, no differences in EC50 were identified between healthy volunteers and hyperuricemic patients. For oxypurinol, only studies in hyperuricemic patients (RDEA3170-107 and RDEA3170-206) were available for model building, therefore, potential differences could not be assessed.

Study D5495C00006, a multiple dose study in healthy Asian volunteers treated with either 12 mg or 24 mg verinurad dose combined with 300 mg allopurinol, was not included in the modeling, and was instead used as a validation study. The model was found to predict the steady-state data from this study well, as judged by visual predictive check plots (see Supplementary Material). It should be acknowledged that one limitation of the validation is that study D5495C00006 did not include measurements of uric acid at an untreated baseline.

### Temporal uric acid response to verinurad in combination with febuxostat

**Fig. 2:**
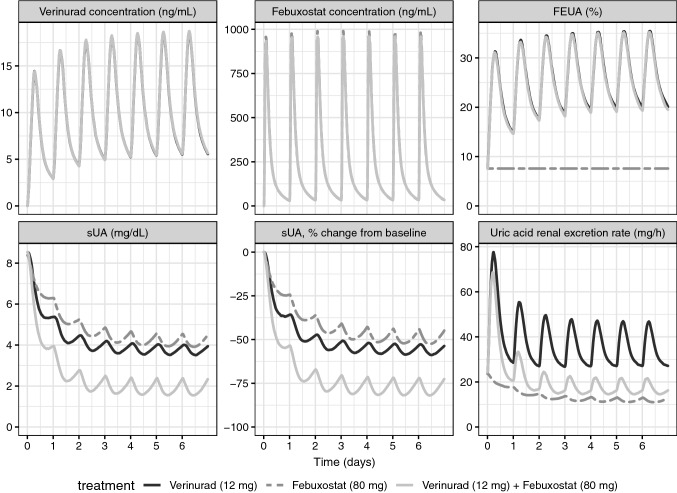
Time profiles of model-predicted verinurad (ER8 formulation) and febuxostat concentration, FEUA, sUA reduction and uric acid renal excretion rate. Simulation with variability (see “Methods” section for details). Simulations were performed assuming following baseline characteristics: eGFR = 60 mL/min/1.73 m^2^; baseline sUA = 8.5 mg/dL; baseline FEUA = 7.7%; non-Asian; verinurad ER8 formulation given in fed state. *FEUA* fractional excretion of uric acid, *sUA* serum uric acid

Figure 2 shows model simulations for 1-week treatment with once-daily dosing of either verinurad alone (12 mg dose of ER8 formulation), febuxostat alone (80 mg), and a combination of two drugs (12 mg verinurad and 80 mg febuxostat), for a typical patient with an eGFR of 60 mL/min/1.73 m^2^ and baseline sUA of 8.5 mg/dL. The sUA reduction following combination treatment is considerably larger compared with the effect of either of the drugs alone. Verinurad, as a URAT1 inhibitor that promotes renal excretion of uric acid, has a direct effect on FEUA. This is illustrated in the bottom right plot in Fig. 2, where FEUA increases following either verinurad monotherapy or verinurad in combination with XOI. In both scenarios, FEUA can be seen to fluctuate on average between approximately 19.4% and 35.5% during the 24 h interval at steady state (when baseline FEUA is ~ 7.7%). The effect on FEUA is the same regardless of verinurad monotherapy or its combination with a XOI, since a XOI has no impact on FEUA. Daily fluctuations in FEUA following verinurad treatment are accompanied by fluctuations in uric acid renal excretion rate (bottom middle plot). If verinurad is given as monotherapy, renal excretion rate of uric acid is considerably increased. During steady state, it can fluctuate on average between 27.1 and 47.7 mg/h. When verinurad is administered with XOI, renal excretion rate at steady-state is reduced compared with verinurad monotherapy; the daily fluctuations are between 13.8 and 21.3 mg/h and the maximum renal excretion rate at steady state is similar to the value at baseline (approximately 21.3 mg/h compared with 24.3 mg/h, respectively). The full effect of combination treatment is achieved after a few days of administration, once verinurad and XOI exposures have reached steady-state; maximum renal excretion rate is initially increased to approximately 66.2 mg/h on day 1 and this value is reduced to 32.4, 23.8, and 21.9 mg/h on days 2, 3, and 4, respectively. At day 5, the value reaches approximately 21.3 mg/h and continues to stay at this level (bottom middle plot of Fig. 2).

### Dose–response for verinurad in combination with a XOI in CKD and HF patients

**Fig. 3:**
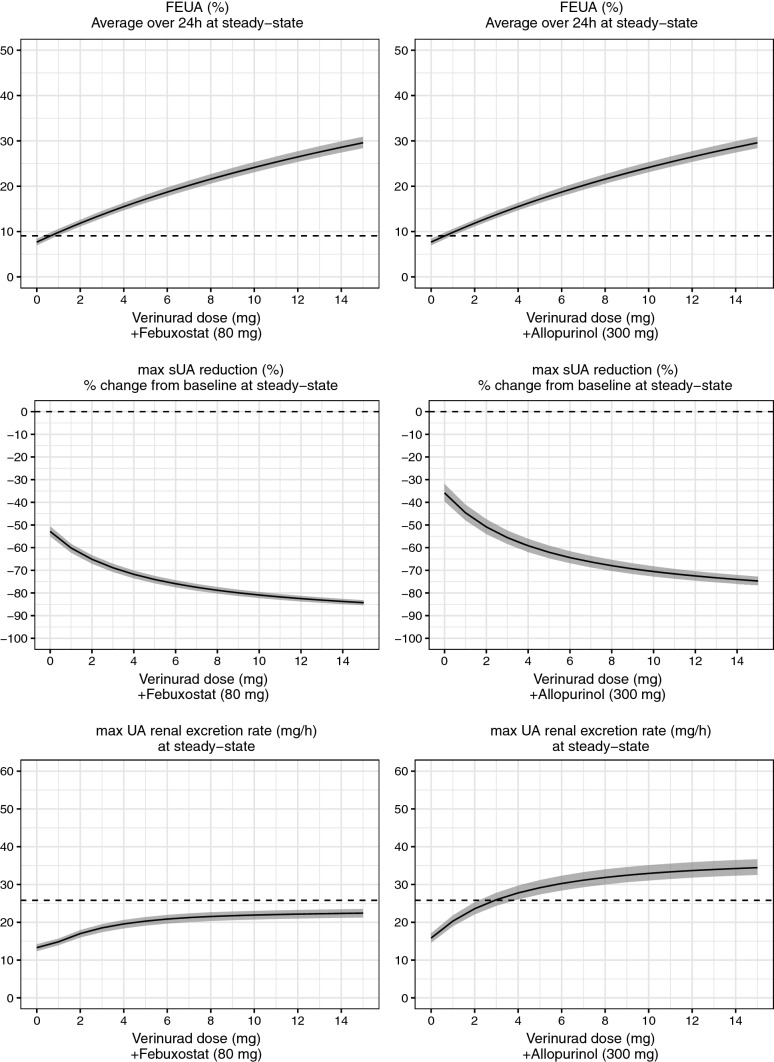
Model-predicted dose–response relationship for steady-state 24 h average FEUA, maximum sUA reduction, and maximum UA renal excretion rate for verinurad (ER8 formulation) in combination with 80 mg febuxostat (left panel) and 300 mg allopurinol (right panel). Simulation with uncertainty (see “Methods” section for details). Solid line and shaded area correspond to median and 5th and 95th quantile; dashed line corresponds to the median placebo response. Simulations were performed assuming following baseline characteristics: eGFR = 60 mL/min/1.73 m^2^; baseline sUA = 8.7 mg/dL; baseline FEUA = 7.7%; non-Asian. *eGFR* estimated glomerular filtration rate, *ER8* extended release formulation, *FEUA* fractional excretion of uric acid, *sUA* serum uric acid

Figure 3 shows model-predicted relationship between verinurad dose and several uric acid responses; 24 h average FEUA, maximum UA renal excretion rate, and maximum sUA reduction. A clear dose–response can be observed for all endpoints. In this simulation, verinurad is always combined with the same XOI dose (here, 80 mg febuxostat or 300 mg allopurinol); this includes dose 0 of verinurad, which consequently corresponds to XOI monotherapy. For reference, placebo response was also added to all plots, to highlight the difference of the verinurad/XOI combination treatment compared with baseline conditions (i.e. no drug).

It should be emphasized that dose–response relationships may be different if alternative baseline patient characteristics are assumed (i.e. baseline eGFR and FEUA). For the simulation presented in Fig. 3, baseline characteristics are used which correspond to patients anticipated in the current CKD and HF development program for verinurad (eGFR of 60 mL/min/1.73 m^2^ and baseline FEUA of 7.7%, which correspond to the population mean FEUA for asymptomatic hyperuricemic patients).

### Application in drug development

**Fig. 4:**
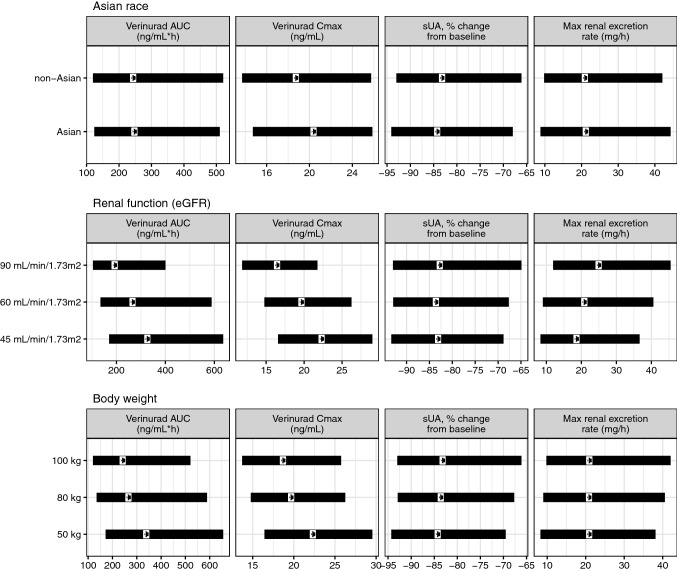
Scenario 1: impact of patient baseline characteristics on verinurad pharmacokinetics (12 mg QD ER8 formulation) (AUC and C_max_) and pharmacodynamic responses to 12 mg verinurad and 80 mg febuxostat (maximum %sUA reduction relative to placebo and maximum renal excretion rate). Simulation with variability (see “Methods” section for details). Dots correspond to median and the range to 5th and 95th quantile. In each panel, a given covariate was compared versus a typical patient using following typical values: eGFR of 60 mL/min/1.73 m^2^ and body weight of 80 kg, non-Asian. *AUC* area under the curve, *Cmax* maximum concentration, *eGFR* estimated glomerular filtration rate, *ER8* extended release formulation, *QD* every day, *sUA* serum uric acid

To illustrate how the semi-mechanistic PKPD model can be used in drug development, we provide two scenarios. The first scenario demonstrates how the model can be used to support dose selection for various patient subpopulations. This is illustrated in Fig. 4, where the most relevant PK (ER8 formulation) and PD parameters of verinurad/XOI combination are simulated for a range of patient baseline covariates: renal function (eGFR), body weight, and race (Asian versus non-Asian). It can be seen that verinurad PK (steady-state area under the curve [AUC] and C_max_), efficacy (maximum sUA reduction) and safety (maximum UA renal excretion rate) are similar between different patient subpopulations, and none of the three assessed covariates impact the PK and PD of the verinurad/XOI combination to a clinically relevant extent. Consequently, this supports that no dose adjustment is needed based on renal function, body weight, and race considering effect on maximum sUA reduction and maximum UA renal excretion rate.

**Fig. 5:**
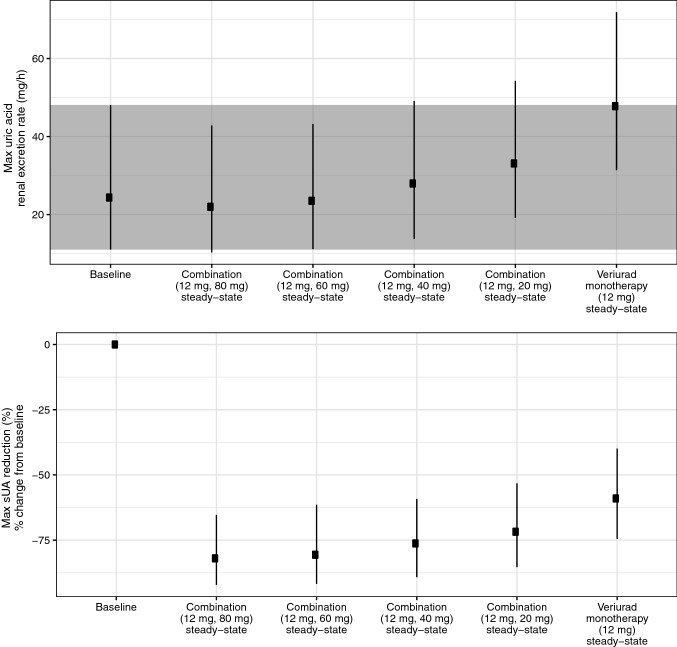
Scenario 2: model-predicted maximum renal excretion of uric acid at steady state and maximal sUA reduction percentage change from baseline at various verinurad (ER8 formulation) and XOI (febuxostat) treatment combinations. Simulation with variability (see “Methods” section for details). Combination doses refer to (verinurad dose, febuxostat dose). Datapoints and error bars correspond to median and 5th and 95th quantile (simulation included 500 subjects); grey area corresponds to the 5th and 95th quantile of the baseline. Simulations were performed assuming following baseline characteristics: eGFR = 60 mL/min/1.73 m^2^; baseline sUA = 8.5 mg/dL; baseline FEUA = 7.7%; non-Asian; verinurad ER8 formulation given in fed state. *eGFR* estimated glomerular filtration rate, *ER8* extended release formulation, *FEUA* fractional excretion of uric acid, *sUA* serum uric acid

The second scenario illustrates simulation of various dose combinations to explore how various XOI doses (here, febuxostat) impact the level of maximum uric acid renal excretion rate, when combined with the same verinurad dose (here, 12 mg of ER8 formulation of verinurad). In this example, the value of using a semi-mechanistic mathematical model is the ability to explore dose combinations that were not tested without having to perform new clinical trials. The results can be seen in Fig. 5 (top panel). The aim of this simulation is to identify which febuxostat dose is sufficient to keep uric acid excretion rate at steady state within safe baseline levels. Combination with febuxostat 60–80 mg gives a comparable level of maximum UA excretion rate at steady-state to baseline. Lower doses of febuxostat, combined with the same 12 mg dose of verinurad, result in an increased maximum UA excretion rate compared with baseline, with the largest increase observed with verinurad monotherapy. According to the simulations, less than 5% of the patients receiving 12 mg verinurad combined with either 60 or 80 mg febuxostat are expected to have a maximum UA excretion rate greater than 95th quantile of the baseline (value indicated by the upper line of the grey area in Fig. 5, approximately 48 mg/h). In contrast, nearly 50% of patients receiving 12 mg verinurad monotherapy are predicted to have their maximum UA excretion levels above this value.

At the same time, the model can be used to assess the efficacy of the simulated combinations, by predicting the impact on maximum sUA reduction (Fig. 5, bottom panel). This is valuable when determining the optimal doses of the combination, by providing an estimate of safety (maximum uUA excretion) and efficacy (sUA reduction) for each dose combination.

## Discussion

The semi-mechanistic exposure–response model was successfully used to describe data from several phase 1 and phase 2a studies in the clinical development program for verinurad. In the work presented here, an existing uric acid model structure was used as a starting point for model development [23]. To extend the model, interindividual variability was included to explore the impact of individual covariates on the pharmacodynamics and safety of verinurad.

The parameter estimates related to uric acid dynamics were similar as reported previously [23], supporting the ability to recover parameters of uric acid disposition equally well with either drug treatment, as both lesinurad and verinurad affect uric acid disposition in the same way through URAT1 inhibition [16]. The potency of verinurad (EC50) was found to be somewhat higher in hyperuricemic patients, compared with healthy volunteers, in line with the results shown for lesinurad [23]. In the current model, febuxostat potency (EC50) was not found to be significantly different between healthy subjects and hyperuricemic patients, even though this was reported previously [23].

### FEUA variability

Baseline FEUA was found to be higher in healthy subjects compared with patients with hyperuricemia, in agreement with previous reports [33]. In addition, we showed a difference in FEUA between two types of hyperuricemic patients: symptomatic (gout) and asymptomatic (patients included in D5495C00001 study), with symptomatic patients having the lowest FEUA value. Interestingly, Asian subjects were found to have somewhat lower FEUA compared with non-Asians; however genetic data are needed to unequivocally attribute this effect to Asian race, such as higher prevalence in individuals of Asian descent of certain polymorphisms in the URAT1 gene. Polymorphisms in the URAT1 gene have been linked to hyperuricemia in Asian populations, although further elucidation of the precise mechanism of the effect of these polymorphisms on URAT1 function is required [34, 35]. If confirmed, lower FEUA may be due to a slightly higher eGFR in the Asian subjects in our studies (thus Asian race may confound the effect of eGFR explicitly included in our model) or different purine content in the Asian diet.

### Applications of this model

The strength of this uric acid model is the ability to simultaneously describe two different endpoints: sUA reduction (related to efficacy) and uUA excretion (related to safety). This is valuable when selecting an optimal dose during drug development, as it allows a quantitative assessment of the balance between efficacy and safety. A safety concern with URAT1 monotherapy is serum creatinine elevation, which has been partly attributed to an increased urinary renal excretion rate of uric acid leading to its precipitation and microcrystallization in renal tubules [22]. To mitigate this risk, verinurad is always combined with a XOI, leading to a reduction in renal excretion rate. With a semi-mechanistic model, which can quantitatively describe serum acid dynamics, it is possible to simulate various scenarios. For example, different dose combinations (including those not tested in a clinical setting) of verinurad and XOI can be simulated to identify the optimal dosing regimen, e.g. one that brings uUA excretion to baseline (as illustrated in Fig. 5).

Another valuable application of this model is the opportunity to predict maximum sUA reduction and maximum uUA excretion for individual subjects in phase 2 and phase 3 studies using sUA and uUA data, even if the data is sparse. Timing these observations without modeling maxima of serum and urine UA is challenging in such a setting (i.e. large trials). For example, maximum reduction of uric acid occurs rather late in the day (if the dose is taken in the morning), as observed in simulated time profiles in Fig. 2. Therefore, sample collection would ideally need to take place in the afternoon, which may often not be feasible. Additionally, there remains some discussion regarding whether maximal uric acid excretion rate is a predictor of renal safety; a surge in uUA excretion may lead to crystallization and subsequent tubular luminal obstruction, resulting in acute kidney injury [36]. Currently, the likelihood of uric acid crystallization is reduced through increasing fluid intake [37] and measurement of maximal uric acid excretion rate requires collection of urine throughout the day in regular time intervals, which is difficult to obtain in large phase 2 or 3 studies.

### Impact of covariates

The exploration of covariate impact on verinurad and XOI treatment showed that none of the covariates investigated had a clinically relevant impact on efficacy and safety (as demonstrated in Fig. 4 for the combination of 12 mg verinurad and 80 mg febuxostat). This supports the use of the same doses of verinurad and XOI given in combination for all patients, regardless of renal function, body weight and race (Asian vs non-Asian) in the current development program. The similar reduction in sUA from baseline with the same dose of verinurad for low and normal renal function is related to the increase in UA renal clearance with higher verinurad exposure. This increase with higher verinurad exposure is counteracted by decreased renal clearance due to lower eGFR.

### Limitations

It should be highlighted that the conclusions of the model and subsequent dose recommendations apply to patients with renal function and body weight within the range studied here and it cannot be excluded that PK and/or PD may be affected to a clinically relevant extent in patients with different characteristics. For example, one limitation of the current work is the inclusion of a limited number of patients with severe renal impairment (eGFR < 30 mL/min/1.73 m^2^). Therefore, this model may not reflect the exposure–response relationship in patients with severely impaired kidney function, as our current model overpredicts sUA and uUA in the renal impairment study RDEA3170-108 [38]. One explanation for this overprediction may be a higher FEUA in renally impaired patients, which in turn affects sUA level and the effect of the treatment. However, URAT1 inhibition becomes ineffective at very low eGFR, and therefore patients with eGFR below 25 ml/min/1.73 m^2^ are not included in the ongoing phase 2b studies. Examining the assumptions of this model with regards to the effects of renal impairment, such as the assumption that renal impairment is reflected only in a decreased eGFR (and FEUA is unchanged), may be one direction for evolving the model in the future. A phase 2b study of verinurad in patients with CKD (NCT03990363) will provide further data on URAT1 inhibition and the effects of renal impairment in this patient population.

Overestimation of urinary UA data for some of the studies is seen in subjects who have the highest amounts of cumulative urinary UA. This corresponds to the long urine collection interval overnight, suggesting that the rate of uUA excretion is not constant over the collection period. This may need to be examined further and incorporated into future models. Additionally, the ability to accurately describe the dose–response component of allopurinol is limited due to data availability; the only doses of allopurinol studied in the verinurad clinical program are 300 mg and 600 mg, both of which result in close to maximum effect on sUA reduction. Although the model can describe the steady-state effect of 300 mg allopurinol adequately, the extrapolation to lower doses may not be reliable.

### Benefits of verinurad

While comparison of verinurad and lesinurad are out of scope of this paper, we note that verinurad can be predicted to have improved serum UA lowering and reduced urine UA excretion compared with lesinurad for two reasons: greater potency of verinurad for the URAT1 transporter and slow absorption when given in the extended release formulation [16]. Firstly, higher potency allows for low doses of verinurad to be used clinically. Secondly, reducing the absorption rate of verinurad as a strategy to reduce potential renal-damaging effects of UA excretion, while still maintaining serum UA lowering, is based on no change to the AUC at steady state while reducing Cmax. Lower Cmax reduces the maximum UA urinary excretion rate compared with the immediate release formulation. The maintained AUC of verinurad drives continuing excretion of UA and maintains serum UA lowering.

### Adherence

Modelling of dual urate-lowering therapy has suggested that poor adherence to therapy may negatively impact the efficacy and safety profiles; when dosing is restarted following missed doses, uUA excretion is increased [39]. A fixed-dose combination has been developed, so that patients will always receive both the URAT1 inhibitor and the XOI.

### Future expansions of the model

It is worth mentioning that sUA levels fluctuate during 24 h at baseline, i.e. without any drug treatment. This may indicate the presence of circadian rhythm and/or impact of diet, which has been previously reported in literature [40]. The assessment of sUA circadian rhythm was not within the scope of the current work, and could be an expansion of the current model. Additional information may be required to accurately describe such daily sUA variation, for example differences in food intake that may affect purine metabolism.

## Conclusions

The semi-mechanistic exposure–response model for verinurad combined with either febuxostat or allopurinol was successfully developed and described the data well, taking into account impact of various patient characteristics, such as renal function, baseline fractional excretion of uric acid, and race. Simulations demonstrate how this model can be used to justify dose selection in patient subpopulations. The model can support clinical development by simulating different scenarios with respect to treatment and/or patient populations.

## Supplementary Information

Below is the link to the electronic supplementary material.

Supplementary file1 (DOCX 997 kb)

Supplementary file2 (DOCX 572 kb)

Supplementary file3 (DOCX 792 kb)

Supplementary file4 (DOCX 1142 kb)
