# Supplementary material for: A semi-mechanistic exposure–response model to assess the effects of verinurad, a potent URAT1 inhibitor, on serum and urine uric acid in patients with hyperuricemia-associated diseases
Source: J Pharmacokinet Pharmacodyn. 2021 Mar 17;48(4):525–41. doi: 10.1007/s10928-021-09747-y (PMC8225519; doi:10.1007/s10928-021-09747-y)
Supplement: Supplementary file 1 — Supplementary file1 (DOCX 997 kb) [file 10928_2021_9747_MOESM1_ESM.docx]

A semi-mechanistic exposure-response model to assess the effects of verinurad, a potent URAT1 inhibitor, on serum and urine uric acid in patients with hyperuricemia-associated diseases

**Jacob Leander^1^, Mikael Sunnåker^1^, Dinko Rekić^1^, Sergey Aksenov^2^, Ulf G. Eriksson^1^, Susanne Johansson^1^, Joanna Parkinson^1^**

*^1^Clinical Pharmacology and Quantitative Pharmacology, Clinical Pharmacology and Safety Sciences, R&D, AstraZeneca, Gothenburg, Sweden*

*^2^Clinical Pharmacology and Quantitative Pharmacology, Clinical Pharmacology and Safety Sciences, R&D, AstraZeneca, Waltham*

**Corresponding author**

Joanna Parkinson

email: Joanna.parkinson@astrazeneca.com

# Online Resource 1. Population pharmacokinetic model for verinurad modified release 4 (MR4) formulation

A population pharmacokinetic (popPK) model for the modified release 4 (MR4) verinurad formulation was developed using data from eight studies: RDEA3170-104, RDEA3170-105, RDEA3170-107, RDEA3170-108, RDEA3170-110, RDEA3170-204, RDEA3170-205, and RDEA3170-206. Details of all studies can be found in Table 1. PK sampling schedules are presented in Table 2. A summary of the demographics and baseline characteristics of the subjects included in the analysis can be found in Table 3. Exploratory plots of the observed pharmacokinetic data from each study can be found in Fig. 1.

In total, 9439 records of verinurad concentrations in a total of 286 subjects were available. The popPK model of verinurad MR4 was modeled as a linear two-compartmental disposition model. The absorption model required a model with a combination, a zero-order duration into depot, and a first-order absorption model from the depot to central compartment.

The error model was described as additive on log scale. Variability across individuals was modelled as a log-normal distribution on central clearance, peripheral volume, zero-order duration, first-order absorption, and relative bioavailability.

Continuous covariates were incorporated using a power model. Estimated glomerular filtration rate (eGFR) was found to be a significant covariate on verinurad apparent clearance, with a higher eGFR associated with a higher apparent clearance. Body weight was found to be a significant covariate for the central and peripheral volumes as well as for the central and inter-compartmental clearances.

Discrete covariates were coded using multiplicative factors, where the typical value was multiplied by (1+THETA). Food status was found to be a significant covariate on the bioavailability and zero order duration. Asian race was found to be a significant covariate on the relative bioavailability.

Parameter estimates from the final model can be found in Table 4.

In Fig. 2, a standard panel of goodness-of-fit plots for the MR4 popPK model is shown. In Fig. 3, a prediction-corrected VPC for the MR4 popPK model is shown. The model diagnostics shows that the pharmacokinetic data of verinurad MR4 formulation is adequately described using the final model.

Table 1. Summary of clinical studies included in verinurad MR4 popPK modeling

| Study | Description | Population | Active treatments |
| --- | --- | --- | --- |
| RDEA3170-104 | Single and multiple dose study in Japanese subjects | Healthy volunteers | 2.5 mg verinurad  5 mg verinurad  10 mg verinurad  15 mg verinurad |
| RDEA3170-105 | Verinurad and febuxostat drug interaction study | Healthy volunteers | 2.5 or 10 mg verinurad  40 mg febuxostat  40 mg febuxostat + 10 mg verinurad  80 mg febuxostat  80 mg febuxostat + 2.5 mg verinurad |
| RDEA3170-107 | Verinurad and allopurinol combination study in gout subjects | Symptomatic hyperuricemic | 10 mg verinurad  300 mg allopurinol  300 mg allopurinol + 10 mg verinurad |
| RDEA3170-108 | PK renal impairment study | Healthy volunteers | 15 mg verinurad |
| RDEA3170-110 | Bioavailability study | Healthy volunteers | 10 mg verinurad |
| RDEA3170-204 | Phase IIa verinurad and febuxostat combination study in gout subjects | Symptomatic hyperuricemic | 40 mg febuxostat  40 mg febuxostat + 2.5, 5, 10, 15, or 20 mg verinurad  80 mg febuxostat  80 mg febuxostat + 2.5, 5, 10, or 15 mg verinurad |
| RDEA3170-205 | Phase IIa verinurad and febuxostat combination study in Japanese gout subjects | Symptomatic hyperuricemic | 2.5, 5, 10, or 15 mg verinurad  10, 20, or 40 mg febuxostat  10 mg febuxostat + 2.5 or 5 mg verinurad  20 mg febuxostat + 2.5, 5, or 10 mg verinurad  40 mg febuxostat + 5 or 10 mg verinurad |
| RDEA3170-206 | Phase IIa verinurad and allopurinol combination study in gout subjects | Symptomatic hyperuricemic | 300 mg od allopurinol  600 mg od allopurinol  300 mg bid allopurinol  300 mg od allopurinol + 2.5, 5, 7.5, 10, 15, or 20 mg verinurad |

bid, twice daily; MR4, modified release 4; od, once daily; popPK, population pharmacokinetic.

Table 2. Schedules for PK, sUA and uUA sampling for the studies included in the analysis

| **Study** | **PK sampling schedule** | **sUA sampling schedule** | **uUA sampling schedule** |
| --- | --- | --- | --- |
| RDEA3170-104 | - Days 1 and 12: predose^*^, and 15, 30, and 45 min, and 1, 1.5, 2, 2.5, 3, 4, 5, 6, 8, 10, 12, 24, 30, 36, 48, 54, 60, and 72 h postdose - Day 6: predose^*^ and 15, 30, and 45 min, and 1, 1.5, 2, 2.5, 3, 4, 5, 6, 8, 10, 12, and 24 h postdose - Days 8 to 11: predose^*^ | - Day 1: –24, –21, –18, –12 h prior to dosing on Day 1, predose^*^, and 3, 6, 12, 24, 30, 36, 48, 54, 60, and 72 h postdose - Day 6: –24, –21, –18, –12 h prior to dosing on Day 6, predose^*^, and 3, 6, 12, and 24 h postdose - Days 8 to 11: predose^*^ - Day 12: predose^*^, and 3, 6, 12, 24, 30, 36, 48, 54, 60, and 72 h postdose | - Day 1: –24 to –18, –18 to –12, and –12 to 0 h prior to dosing on Day 1, and 0 to 6, 6 to 12, 12 to 24, 24 to 30, 30 to 36, 36 to 48, 48 to 60, and 60 to 72 h post-dose - Day 6: -24 to -18, -18 to -12, and -12 to 0 h prior to dosing on Day 6, and 0 to 6, 6 to 12, and 12 to 24 h postdose. - Day 12: 0 to 6, 6 to 12, 12 to 24, 24 to 30, 30 to 36, 36 to 48, 48 to 60, and 60 to 72 h postdose. |
| RDEA3170-105 | - Days 7, 14, and 21: predose^*^ and at 15, 30, and 45 min and 1, 1.5, 2, 2.5, 3, 4, 5, 6, 8, 10, 12, and 24 h postdose | - Day –1: –24, –21, –18, and –12 h - Day 1: predose^*^ - Days 7, 14, and 21: predose^*^ and 3, 6, 12 and 24 h postdose | - Day –1: –24 to –18, –18 to –12, and –12 to  0 h - Days 7, 14, and 21: 0 to 6, 6 to 12, and 12 to 24 h postdose |
| RDEA3170-107 | - Days 7, 14, and 21: predose and 1, 2, 3, 4, 5, 6, 12, 22, and 24 h postdose | - Day –1: –24, –23, –22, –21, –20, –19, –18, –12, and -2 h prior to dosing on Day 1 and at predose - Days 7, 14, and 21: predose and 1, 2, 3, 4, 5, 6, 12, 22, and 24 h postdose | - Day –1: –24 to –23, –23 to –22, –22 to –21, –21 to –20, –20 to –19, –19 to –18, –18 to –16, –16 to –14, –14 to –12, –12 to –2, and –2 to 0 h predose - Days 7, 8, 14, and 21: 0 to 1, 1 to 2, 2 to 3, 3 to 4, 4 to 5, 5 to 6, 6 to 8, 8 to 10, 10 to 12, 12 to 22, and 22 to 24 h postdose |
| RDEA3170-108 | - Predose^*^ and 30 min, 1, 1.5, 2, 3, 4, 5, 6, 8, 10, 12, 16, 24, 30, 36, 48, 54, 60, and 72 h postdose | - –24, –21, –18, and –12 h predose - Within 30 min predose and at 3, 6, 12, 24, 30, 36, 48, 54, 60, and 72 h postdose. | - Predose: –24 to –18, –18 to –12, and –12 to 0 h - Postdose: 0 to 6, 6 to 12, 12 to 24, 24 to 36, 36 to 48, 48 to 60, and 60 to 72 h |
| RDEA3170-110 | - Days 1, 5, 9 and 13: predose^*^, 30 min and 1, 1.5, 2, 3, 4, 6, 8, 10, 12, 24, 36, 48, and 72 h postdose. | - Day –1: –24, –23, –22, –21, –20, –18, –16, –14, and –12 h predose - Days 1, 5, 9, and 13: predose^*^ and 1, 2, 3, 4, 6, 8, 10, 12, and 24 h postdose | - Day –1: –24 to –21, –21 to –18, –18 to –12, and –12 to 0 h predose - Days 1, 5, 9, and 13: 0 to 3, 3 to 6, 6 to 12, and 12 to 24 h postdose |
| RDEA3170-204 | - Days 7, 14, 21, and 28: predose^*^ and 30 min, 1, 1.5, 2, 3, 4, 6, 8, 10, 12, 22, and 24 h postdose | - In relation to RDEA3170 and/ or febuxostat dosing on Day 1: Day –1 (–24, –23, –22,  –21, –20, –19, –18, –16, –14, –12), and –2 h prior to dosing on Day 1, and at predose^*^ - In relation to RDEA3170 and/or febuxostat dosing on Days 7, 14, 21, and 28: predose^*^ and 1, 2, 3, 4, 5, 6, 8, 10, 12, 22, and 24 h postdose | - In relation to RDEA3170 and/or febuxostat dosing on Day 1: Day –1 (–24 to –23, –23 to –22, –22 to –21, –21 to –20, –20 to –19, –19 to –18, –18 to –16, –16 to –14, –14 to –12, –12 to –2), and –2 to 0 h predose - In relation to RDEA3170 and/or febuxostat dosing on Days 1 (Cohort 5 only), 7, 14, 21, and 28: 0 to 1, 1 to 2, 2 to 3, 3 to 4, 4 to 5, 5 to 6, 6 to 8, 8 to 10, 10 to 12, 12 to 22, and 22 to 24 h postdose |
| RDEA3170-205 | - In relation to RDEA3170 and/or febuxostat dosing on Day 1: 30 min, 1, 1.5, 2, 3, 4, 6, 8, 12 and 24 h postdose; and on Days 7, 14, 21, and 28 at predose^*^ and 30 min, 1, 1.5, 2, 3, 4, 6, 8, 10, 12, and 24 h postdose. | - Day 1: –2 h and predose^*^ and 1, 2, 3, 4, 5, 6, 8, 12 and 24 h postdose - Days 7, 14, 21 and 28: predose^*^ and 1, 2, 3, 4, 5, 6, 8, 12 and 24 h postdose | - Day –1: –12 to -2 h - Day 1: –2 to 0 h predose, 0 to 1, 1 to 2, 2 to 3, 3 to 4, 4 to 5, 5 to 6, 6 to 8, 8 to 10, 10 to 12 and 12 to 22, 22 to 24 h postdose - Day 7, 14, 21, 28: 0 to 1, 1 to 2, 2 to 3, 3 to 4, 4 to 5, 5 to 6, 6 to 8, 8 to 10, 10 to 12 and 12 to 22, 22 to 24 h postdose |
| RDEA3170-206 | - Days 7, 14, 21, 28, and 35: predose^*^ and 1, 2, 3, 4, 5, 6, 8, 10, 12, 22, and 24 h postdose | - Day –1: –24, –23, –22, –21, –20, –19, –18, –16, –14, –12, and –2 h prior to dosing on Day 1, and at predose^*^ - Days 7, 14, 21, 28, and 35: predose and 1, 2, 3, 4, 5, 6, 8, 10, 12, 22, and 24 h postdose | -^†^ |

*within 30 min prior to dosing. PK, pharmacokinetic; sUA, serum uric acid; uUA, urinary uric acid

Table 3. Summary of baseline characteristics of patients included in the analysis

| **Study** | **n** | **Age (years) median (min, max)** | **Body weight (kg) median (min, max)** | **eGFR (mL/min/1.73 m^2^) median (min, max)** | **Sex (Males) n (%)** | **Race: Caucasian n (%)** | **Race: Black n (%)** | **Race: Asian n (%)** | **Race: Other n (%)** |
| --- | --- | --- | --- | --- | --- | --- | --- | --- | --- |
| RDEA3170-104 | 36 | 33.5 (21, 53) | 71.9 (54.9, 101.7) | 112.3 (78, 138.3) | 36 (100) | 3 (8.3) | 3 (8.3) | 30 (83.3) | 0 (0) |
| RDEA3170-105 | 20 | 35 (21, 48) | 87.95 (65.8, 115.9) | 97.7 (75.7, 117.7) | 20 (100) | 13 (65) | 6 (30) | 0 (0) | 1 (5) |
| RDEA3170-107 | 12 | 51.5 (29, 69) | 99.7 (80.9, 126.3) | 84.3 (54.1, 102.9) | 12 (100) | 7 (58.3) | 4 (33.3) | 0 (0) | 1 (8.3) |
| RDEA3170-108 | 31 | 61 (35, 81) | 82.6 (59, 122) | 74 (12.3, 106.1) | 31 (100) | 23 (74.2) | 7 (22.6) | 0 (0) | 1 (3.2) |
| RDEA3170-110 | 15 | 44 (25, 62) | 90.9 (65.4, 129.4) | 99.2 (74.9, 134.3) | 15 (100) | 11 (73.3) | 2 (13.3) | 0 (0) | 2 (13.3) |
| RDEA3170-204 | 60 | 50 (29, 71) | 98.95 (63.8, 137.6) | 90.1 (57.4, 121.1) | 60 (100) | 40 (66.7) | 7 (11.7) | 13 (21.7) | 0 (0) |
| RDEA3170-205 | 72 | 46 (21, 67) | 75.45 (58.3, 115.6) | 105.6 (76.8, 133.2) | 72 (100) | 0 (0) | 0 (0) | 72 (100) | 0 (0) |
| RDEA3170-206 | 40 | 48 (28, 74) | 95.5 (63.1, 147.6) | 89.9 (55.1, 125.6) | 39 (97.5) | 30 (75) | 6 (15) | 4 (10) | 0 (0) |
| All subjects | 286 | 46.5 (21, 81) | 83.55 (54.9, 147.6) | 97.6 (12.3, 138.3) | 285 (99.7) | 127 (44.4) | 35 (12.2) | 119 (41.6) | 5 (1.7) |

eGFR, estimated glomerular filtration rate.

Table 4. Parameter estimates of the verinurad MR4 popPK model

| **Parameter** | **Estimate** | **RSE, %** | **IIV (CV%)** | **RSE, %** |
| --- | --- | --- | --- | --- |
| Central clearance (L/h) | 94.69 | 3.40 | 26.17 | 6.38 |
| Central volume of distribution (L) | 448.6 | 11.0 |  |  |
| Inter-compartmental clearance (L/h) | 144.6 | 5.71 |  |  |
| Peripheral volume of distribution (L) | 919.8 | 6.53 | 58.80 | 7.63 |
| Absorption rate constant (1/h) | 0.7885 | 14.4 | 49.39 | 13.3 |
| Zero-order duration, fasted state (h) | 0.4044 | 7.16 | 50.93 | 6.49 |
| Additive error (log scale) | 0.5153 | 0.328 |  |  |
| Relative bioavailability, F1 | 1 (fixed) | - | 24.75 | 7.09 |
| Asian multiplicative factor, F1 | 0.3537 | 18.8 |  |  |
| eGFR ~ central clearance | 0.5095 | 11.0 |  |  |
| Food multiplicative factor, D1 | 5.816 | 3.92 |  |  |
| Food multiplicative factor, F1 | 0.0986 | 10.8 |  |  |
| BW ~ central/compartmental clearance | 0.6301 | 19.4 |  |  |
| BW ~ central/peripheral volume | 0.7893 | 19.4 |  |  |

BW, body weight; eGFR, estimated glomerular filtration rate; D1, zero order duration into depot compartment; IIV, interindividual variability; MR4, modified release 4; popPK, population pharmacokinetic; RSE, relative standard error.

Fig. 1. Observed pharmacokinetic data for the verinurad MR4 formulation, stratified by study and colored by dose.
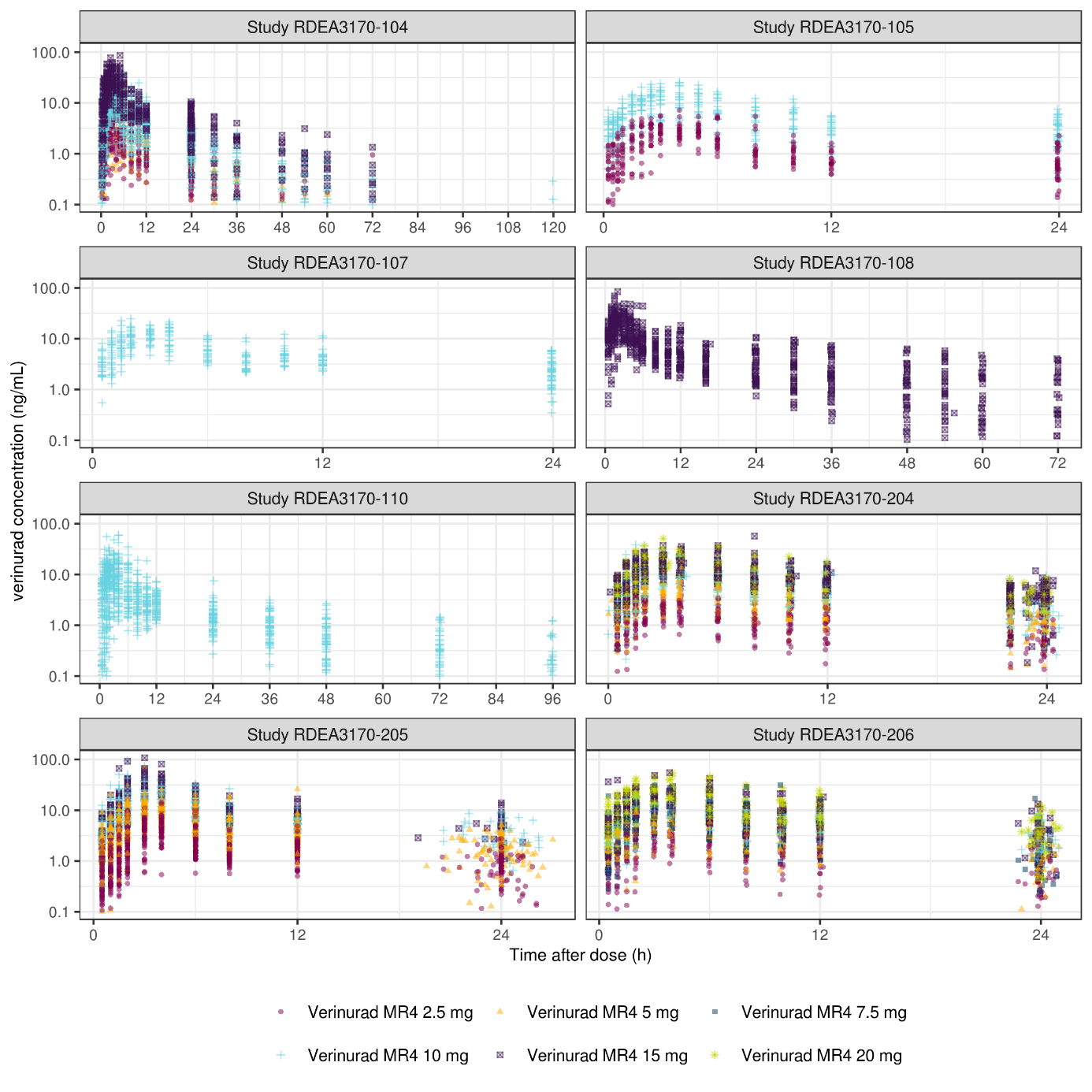


MR4, modified release 4.

Fig. 2. Goodness-of-fit plots for the final verinurad MR4 popPK model. Red line is the line of unity (top-left and middle, and bottom-right), or reference line of zero (bottom-left and middle), or normal distribution with mean of zero and estimated residual variance (top-right). Blue line is the non-parametric smoother (left and middle) or smoothed density line of the residuals (top-right).


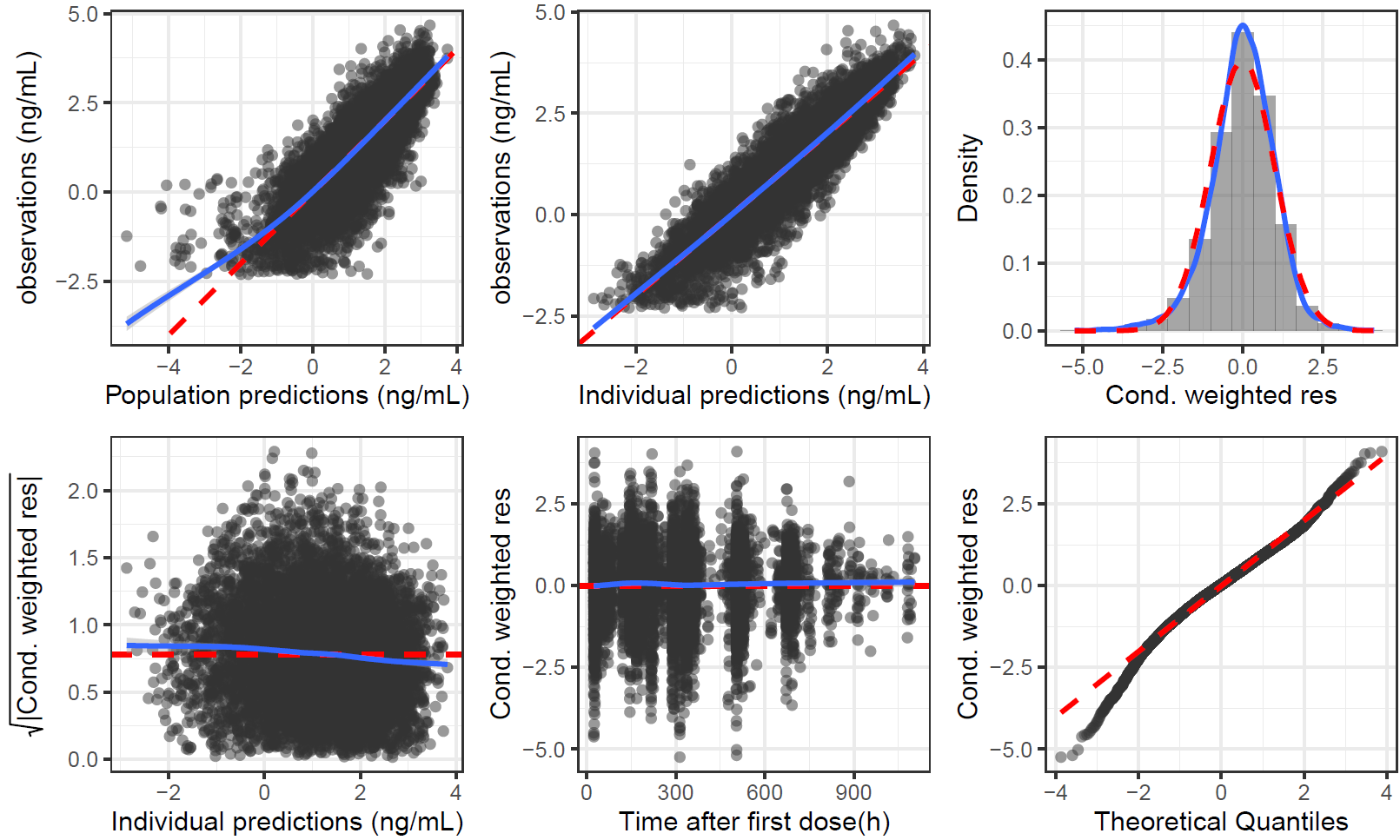


MR4, modified release 4; popPK, population pharmacokinetic.

Fig. 3. Prediction-corrected visual predictive check plot for the final verinurad MR4 popPK model.


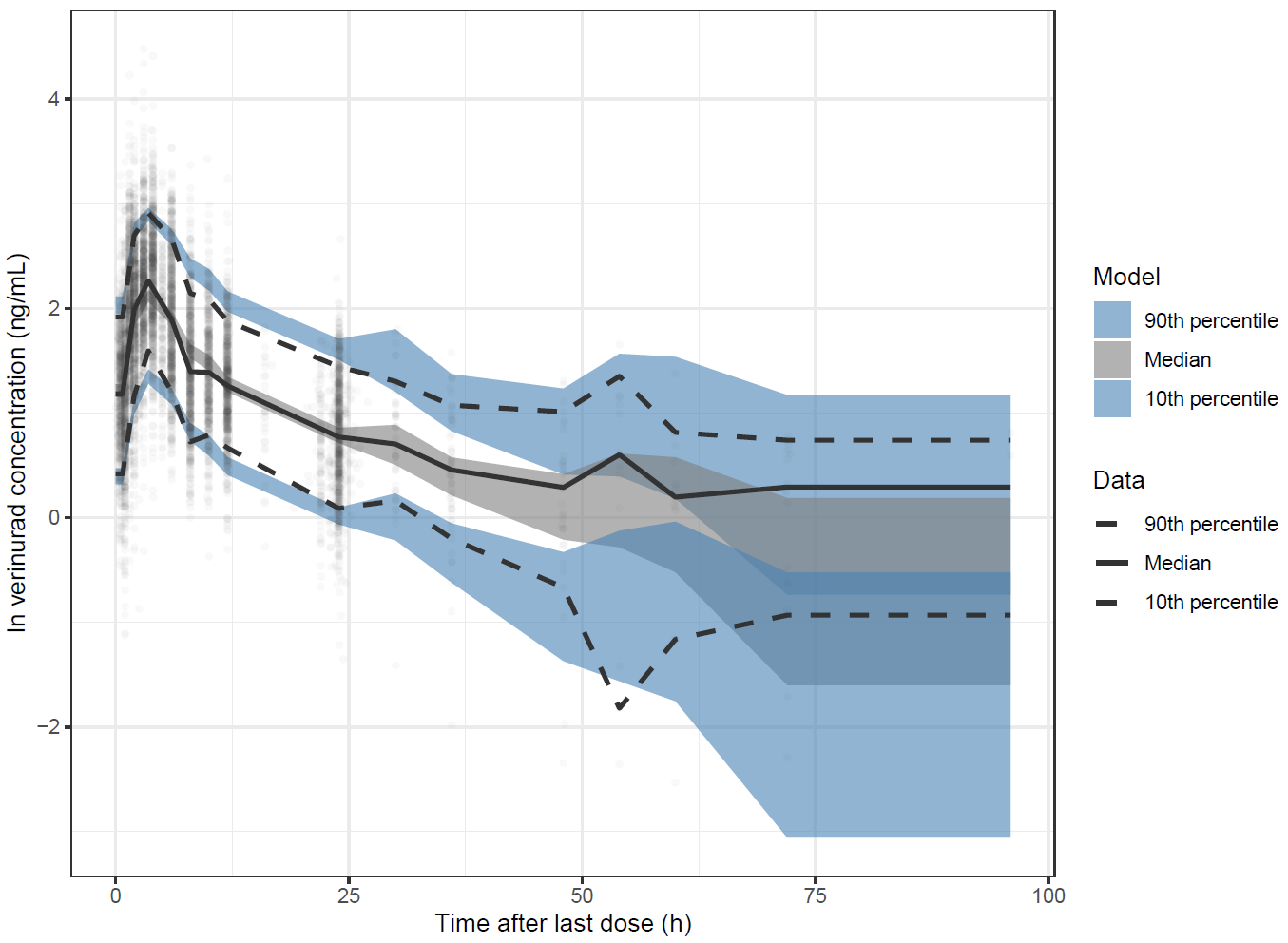


MR4, modified release 4; popPK, population pharmacokinetic.
