# Supplementary material for: A semi-mechanistic exposure–response model to assess the effects of verinurad, a potent URAT1 inhibitor, on serum and urine uric acid in patients with hyperuricemia-associated diseases
Source: J Pharmacokinet Pharmacodyn. 2021 Mar 17;48(4):525–41. doi: 10.1007/s10928-021-09747-y (PMC8225519; doi:10.1007/s10928-021-09747-y)
Supplement: Supplementary file 2 — Supplementary file2 (DOCX 572 kb) [file 10928_2021_9747_MOESM2_ESM.docx]

A semi-mechanistic exposure-response model to assess the effects of verinurad, a potent URAT1 inhibitor, on serum and urine uric acid in patients with hyperuricemia-associated diseases

**Jacob Leander^1^, Mikael Sunnåker^1^, Dinko Rekić^1^, Sergey Aksenov^2^, Ulf G. Eriksson^1^, Susanne Johansson^1^, Joanna Parkinson^1^**

*^1^Clinical Pharmacology and Quantitative Pharmacology, Clinical Pharmacology and Safety Sciences, R&D, AstraZeneca, Gothenburg, Sweden*

*^2^Clinical Pharmacology and Quantitative Pharmacology, Clinical Pharmacology and Safety Sciences, R&D, AstraZeneca, Waltham*

**Corresponding author**

Joanna Parkinson

email: Joanna.parkinson@astrazeneca.com

# Supplementary material 2. Population pharmacokinetic model for verinurad extended release 8 (ER8) formulation

A population pharmacokinetic (popPK) model for the extended release (ER8) formulation for verinurad was developed using data from five clinical studies, described in Table 1. For all studies, data for other formulations than ER8 were excluded in the modeling. PK sampling schedules are presented in Table 2 . A summary of the demographics and baseline characteristics of the subjects included in the analysis can be found in Table 3. Exploratory plots of the observed pharmacokinetic data from each study can be found in Fig. 1.

The popPK model of verinurad takes the form of a two-compartmental disposition model. The ER8 formulation required a rather complex absorption model, comprising zero-order infusion into the dosing compartment and sequential first-order absorption. The elimination process was linear with respect to concentrations of verinurad in the central compartment.

The exploratory analysis of data for study 112 revealed a significant effect of food on the absorption process. Therefore, different lengths of the zero-order infusion into the dosing compartment were assumed for food state (fasted/fed) and estimated from the data.

The error model was described as additive on log scale. Variability across individuals was modelled as a log-normal distribution on central clearance, peripheral volume, zero-order duration, and relative bioavailability.

Covariates were included in the model with a stepwise approach in which new covariates were proposed manually based on visual inspection of correlations between the random effect parameters and candidate covariates. The covariates considered during the modeling were body weight, Asian race (vs all other races), age, sex, and baseline estimated glomerular filtration rate [eGFR], as well as the potential effects of studies on parameters. Body weight and baseline eGFR were incorporated on clearance (Table 4). It was also found that Asian race decreases the uptake of verinurad in the peripheral compartment. Asian race had a borderline significant (95% confidence interval: 0.0076–0.2975) effect on the zero-order infusion but was not part of the model.

The model diagnostics below indicate that the pharmacokinetic data of verinurad is well described using the final model (Fig. 2 and Fig. 3).

Intercompartmental clearance and peripheral volume of were significantly reduced in Asians compared to non-Asians, indicating a decreased uptake of verinurad in the peripheral compartment.

Table 1. Overview of clinical studies used for verinurad ER8 popPK modeling

| Study | Description | Population | Number of patients | Doses |
| --- | --- | --- | --- | --- |
| RDEA3170-112 | Single and multiple dose study | Healthy volunteers | Non-Asian:  n = 39  Asian: n = 1 | 4.5, 6, and 12 mg verinurad |
| D5495C00001 | Pharmacodynamic drug-drug interaction study with verinurad, febuxostat, and dapagliflozin | Asymptomatic hyperuricemic | Non-Asian: n = 34  Asian: n = 2 | 9 mg RDEA3170 + 80 mg febuxostat + 10 mg dapagliflozin  9 mg RDEA3170 + 80 mg febuxostat + placebo |
| D5495C00005 | Bioavailability study | Healthy volunteers | Non-Asian:  n = 25 | 12 mg verinurad (ER8)  Data for the A and B capsules not included in the modeling |
| D5495C00006 | Multiple dose study in Asians/Chinese | Healthy volunteers | Asian:  n = 18 | 12 mg verinurad + 300 mg allopurinol  24 mg verinurad + 300 mg allopurinol |
| D5495C00007 | Phase IIa, verinurad and febuxostat in patients with albuminuria | Type 2 diabetes mellitus, asymptomatic hyperuricemic, with albuminuria | Non-Asian:  n = 26  Asian: n = 1 | 9 mg verinurad +  80 mg febuxostat |

ER8, extended release; popPK, population pharmacokinetic.

Table 2. Schedules for PK, sUA and uUA sampling for the studies included in the analysis

| **Study** | **PK sampling schedule** | **sUA sampling schedule** | **uUA sampling schedule** |
| --- | --- | --- | --- |
| RDEA3170-112 | - Part 1: Days 1, 5, and 9 predose^*^ and at 30 min and 1, 1.5, 2, 3, 4, 6, 8, 10, 12, 24, 36, 48, and 72 h postdose - Part 2: Days 1 and 7 predose^*^ and at 30 min and 1, 1.5, 2, 3, 4, 6, 8, 10, 12, and 24 h postdose - Part 3: Days 1 and 8 predose^*^ and at 30 min and 1, 1.5, 2, 3, 4, 6, 8, 10, 12, 24, 36, 48, and 72 h postdose | - Part 1: Day –1 at –24, –23, –22, –21, –20, –18, –16, –14, and –12 h predose; and on Days 1, 5, and 9 predose^*^ and 1, 2, 3, 4, 6, 8, 10, 12, and 24 h postdose - Part 2: Day –1 at –24, –23, –22, –21, –20, –18, –16, –14, and –12 h predose; and on Days 1 and 7 predose^*^ and 1, 2, 3, 4, 6, 8, 10, 12, and 24 h postdose - Part 3: Days –1 and 7 at –24, –23, –22, –21, –20, –18, –16, –14, and –12 h predose; and on Days 1 and 8 predose^*^ and 1, 2, 3, 4, 6, 8, 10, 12, and 24 h postdose | - Part 1: Day –1 at –24 to –21, –21 to –18,  –18 to –12, and –12 to 0 h predose; and on Days 1, 5, and 9 at 0 to 3, 3 to 6, 6 to 12, and 12 to 24 h postdose - Part 2: Day –1 at –24 to –21, –21 to –18, –18 to –12, and –12 to 0 h predose; and on Days 1 and 7 at 0 to 3, 3 to 6, 6 to 12, and 12 to 24 h postdose - Part 3: Days –1 and 7 at –24 to –21, –21 to –18, –18 to –12, and –12 to 0 h predose; and on Days 1 and 8 at 0 to 3, 3 to 6, 6 to 12, and 12 to 24 hours postdose |
| D5495C00001 | - Treatment period 1: day 7 predose and 15, 30 min, 1, 1.5, 2, 3, 4, 8, 12 and 24 h postdose - Treatment period 2: day 7 predose and 15, 30 min, 1, 1.5, 2, 3, 4, 8, 12 and 24 h postdose | - Treatment period 1: Days –1: single assessment matched by time of day; and day 7 predose and 15, 30 min, 1, 1.5, 2, 3, 4, 8, 12 and 24 h postdose - Treatment period 2: days -1, 7 and 23 predose and 15, 30 min, 1, 1.5, 2, 3, 4, 8, 12 and 24 h postdose | - Day –1: –24 to –23, –23 to –22, –22 to  –21, –21 to –20, –20 to –19, –19 to –18,  –18 to –17, –17 to –16, –16 to –15, –15 to –14, –14 to –13, –13 to –12 h, followed by a single 12 h collection from -12 to 0 h. - Day 7: 0 to 1, 1 to 2, 2 to 3, 3 to 4, 4 to 5, 5 to 6, 6 to 7, 7 to 8, 8 to 9, 9 to 10, 10 to 11, 11 to 12, and 12 to 24 h postdose |
| D5495C00005 | - Day 1: predose, 0.5, 1, 1.5, 2, 3, 4, 5, 6, 8, 10 and 12 h postdose - Day 2: 24 and 36 h postdose - Day 3: 48 h postdose Day 4: 72 h postdose | – | – |
| D5495C00006 | - Day 1 and Day 9: predose, 0.5, 1, 1.5, 2, 3, 4, 5, 6, 8, 10, 12 and 24 h postdose - Day 2: 36 h postdose - Days 3 to 8: predose | - Day –1: –24, –21, –18, and –12 h predose - Days 1 and 7: predose, 3, 6, 12, and 24 h postdose | - Day –1: baseline collection of urine: –24 to –22, –22 to –20, –20 to –18, –18 to –16, –16 to –14, –14 to –12 and –12 to 0 h predose - Days 1 and 7: 0 to 2, 2 to 4, 4 to 6, 6 to 8, 8 to 10, 10 to 12, and 12 to 24 h postdose |
| D5495C00007 | - Days: 8, 15, 29 and 85: predose | - Days: 8, 15, 29 and 85: predose | - |

*within 30 min prior to dosing. PK: pharmacokinetic, sUA: serum uric acid, uUA: urinary uric acid

Table 3. Summary of baseline characteristics of patients included in the analysis

| **Study** | **n** | **Age (years) median  (min, max)** | **Body weight (kg) median  (min, max)** | **eGFR (mL/min/1.73 m^2^) median  (min, max)** | **Sex (Males) n (%)** | **Race: Caucasian n (%)** | **Race:  Black n (%)** | **Race:  Asian n (%)** | **Race:  Other n (%)** |
| --- | --- | --- | --- | --- | --- | --- | --- | --- | --- |
| D5495C00001 | 36 | 41 (20, 63) | 85.7  (57.7, 121.3) | 91.4 (52.8, 128) | 35 (97.2) | 14 (38.9) | 17 (47.2) | 2 (5.6) | 3 (8.3) |
| D5495C00005 | 25 | 41 (19, 49) | 75.4  (50, 98.5) | 103.9 (78.1, 124) | 14 (56) | 25 (100) | 0 (0) | 0 (0) | 0 (0) |
| D5495C00006 | 18 | 38 (27, 48) | 71.9  (58.75, 90.75) | 101.9 (70.4, 117.8) | 17 (94.4) | 0 (0) | 0 (0) | 18 (100) | 0 (0) |
| D5495C00007 | 27 | 62 (43, 79) | 91  (62.1, 136.3) | 55.3 (31.6, 109.9) | 19 (70.4) | 20 (74.1) | 5 (18.5) | 1 (3.7) | 1 (3.7) |
| RDEA3170-112 | 40 | 35.5 (24, 56) | 86.1  (59.2, 126.8) | 99.6 (78.1, 131.6) | 40 (100) | 22 (55) | 17 (42.5) | 1 (2.5) | 0 (0) |
| All subjects | 146 | 41 (19, 79) | 84.55  (50, 136.3) | 96.7 (31.6, 131.6) | 125 (85.6) | 81 (55.5) | 39 (26.7) | 22 (15.1) | 4 (2.7) |

eGFR, estimated glomerular filtration rate.

Table 4. Parameter estimates of the final verinurad ER8 popPK model

| **Parameter** | **Estimate** | **RSE, %** | **IIV (CV%)** | **RSE, %** |
| --- | --- | --- | --- | --- |
| Central clearance (L/h) | 64.1 | 3.60 | 25.4 | 10.4 |
| Central volume of distribution (L) | 259 | 4.20 |  |  |
| Inter-compartmental clearance (L/h) | 55.2 | 7.20 |  |  |
| Peripheral volume of distribution (L) | 675 | 7.70 | 43.9 | 15.7 |
| Relative bioavailability (-) | 1 (FIX) |  | 35.0 | 9.00 |
| Absorption rate constant (1/h) | 0.43 | 2.90 |  |  |
| Zero-order duration, fasted state (h) | 3.45 | 2.50 | 28.6 | 9.50 |
| Zero-order duration, fed state (h) | 6.10 | 8.80 | 28.6 | 9.50 |
| Asian ~ inter-compartmental clearance (-) | 0.44 | 13.6 |  |  |
| Asian ~ peripheral volume of distribution  (-) | 0.59 | 19.8 |  |  |
| eGFR ~ central clearance (-) | 0.72 | 15.9 |  |  |
| BW ~ central clearance (-) | 0.40 | 28.5 |  |  |
| Additive error (log-scale) | 0.50 | 2.70 |  |  |

BW, body weight; eGFR, estimated glomerular filtration rate; ER8, extended release, IIV, interindividual variability; popPK, population pharmacokinetics; RSE, relative standard error.

Fig. 1. Observed pharmacokinetic data for the verinurad ER8 formulation, stratified by study.

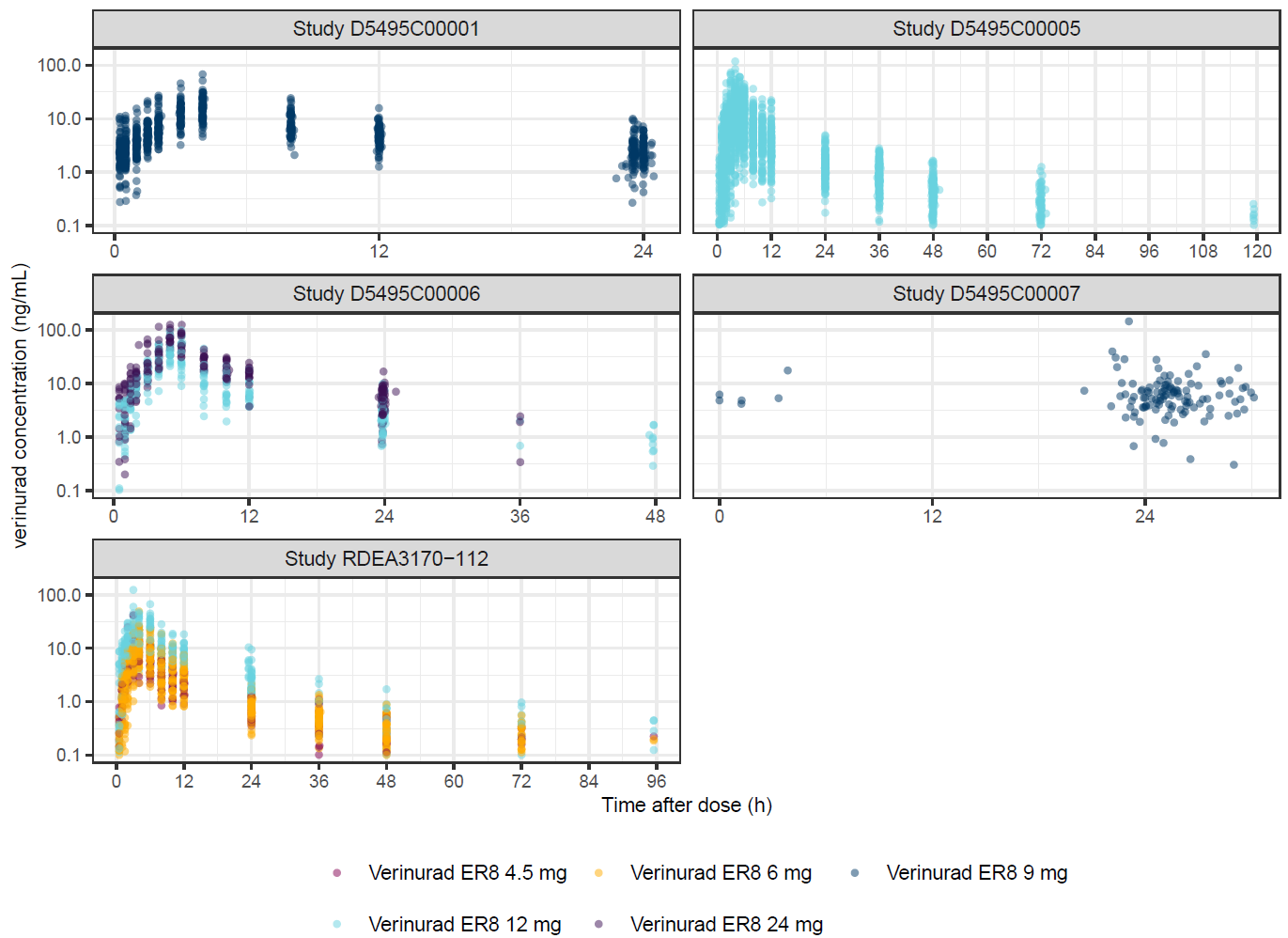


ER8, extended release.

Fig. 2. Goodness-of-fit plots for the final verinurad ER8 popPK model. Red line is the line of unity (top-left and middle, and bottom-right), or reference line of zero (bottom-left and middle), or normal distribution with mean of zero and estimated residual variance (top-right). Blue line is the non-parametric smoother (left and middle) or smoothed density line of the residuals (top-right).


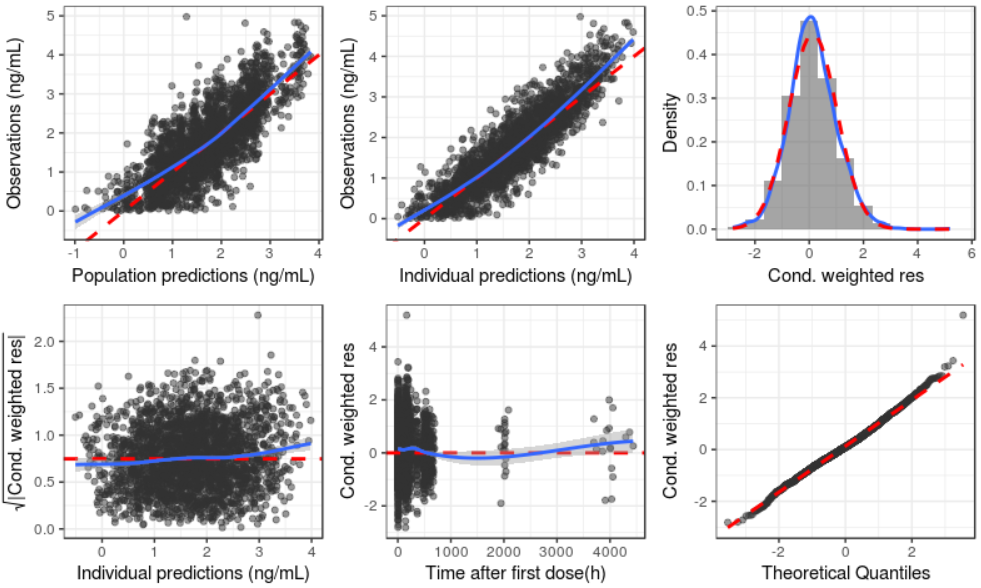


ER8, extended release; popPK, population pharmacokinetic.

Fig. 3. Prediction corrected visual predictive check plot for the final verinurad ER8 popPK model.


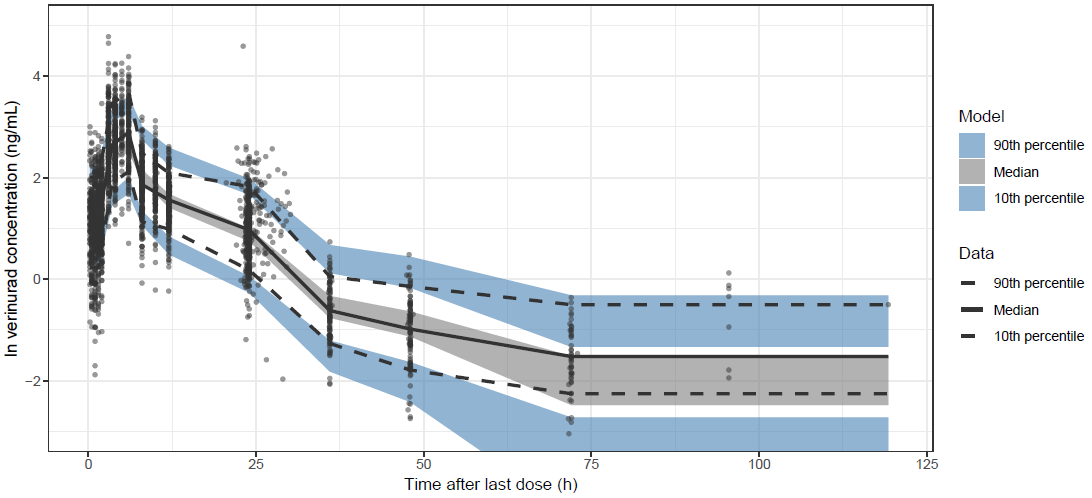


ER8, extended release; popPK, population pharmacokinetic.
