# Supplementary material for: A semi-mechanistic exposure–response model to assess the effects of verinurad, a potent URAT1 inhibitor, on serum and urine uric acid in patients with hyperuricemia-associated diseases
Source: J Pharmacokinet Pharmacodyn. 2021 Mar 17;48(4):525–41. doi: 10.1007/s10928-021-09747-y (PMC8225519; doi:10.1007/s10928-021-09747-y)
Supplement: Supplementary file 3 — Supplementary file3 (DOCX 792 kb) [file 10928_2021_9747_MOESM3_ESM.docx]

A semi-mechanistic exposure-response model to assess the effects of verinurad, a potent URAT1 inhibitor, on serum and urine uric acid in patients with hyperuricemia-associated diseases

**Jacob Leander^1^, Mikael Sunnåker^1^, Dinko Rekić^1^, Sergey Aksenov^2^, Ulf G. Eriksson^1^, Susanne Johansson^1^, Joanna Parkinson^1^**

*^1^Clinical Pharmacology and Quantitative Pharmacology, Clinical Pharmacology and Safety Sciences, R&D, AstraZeneca, Gothenburg, Sweden*

*^2^Clinical Pharmacology and Quantitative Pharmacology, Clinical Pharmacology and Safety Sciences, R&D, AstraZeneca, Waltham*

**Corresponding author**

Joanna Parkinson

email: [Joanna.parkinson@astrazeneca.com](mailto:Joanna.parkinson@astrazeneca.com)

# Online Resource 3. Population pharmacokinetic model for oxypurinol

A population pharmacokinetic (popPK) model for oxypurinol (the active metabolite of allopurinol) was developed using data from three studies: RDEA3170-107, RDEA3170-206, and D5495C00006. Details of all studies can be found in Table 1, while PK sampling schedules are presented in Table 2 A summary of the demographics and baseline characteristics of the subjects included in the analysis can be found in Table 3. Exploratory plots of the observed pharmacokinetic data from each study can be found in Fig. 1.

Only oxypurinol concentration (the active metabolite of allopurinol) was included in the modeling, since it is assumed that it is the concentration of oxypurinol that inhibits uric acid production.

The popPK model of oxypurinol was modeled as a two-compartmental disposition model with first-order absorption. The two-compartmental model structure was selected to achieve flexibility describing PK profiles, given parent allopurinol PK is not modeled, as was also done in Aksenov et al [4], while the absorption model was selected based on Wright et al, who modeled both allopurinol and oxypurinol PK [1]. Our goal was to fit individual subject oxypurinol PK profiles to enable PKPD modeling of uric acid, rather than to simulate uric acid response to allopurinol, given only two doses, 300 and 600 mg were used in the studies in our dataset. The error model was described as additive on a log scale. Variability across individuals was modeled as a log-normal distribution on central clearance.

Estimated glomerular filtration rate (eGFR; higher clearance with higher eGFR) was found to be covariate on oxypurinol clearance, while body weight (higher volume of distribution with higher body weight) was used for oxypurinol central volume of distribution.

It is known that verinurad has an impact on oxypurinol exposure, since oxypurinol, like urate, is a substrate for URAT1 [2]. URAT1 is involved in renal reabsorption of oxypurinol and therefore its renal clearance may be affected by coadministration with URAT1 inhibitors [3]. To account for this effect, an impact of verinurad on oxypurinol clearance was included in the popPK model, in a similar way as previously used for lesinurad pharmacokinetic model (Sergey Aksenov and Hongmei Xu, personal communication):

CL = CLTYP * (1 + log(1+k*DOSE_VERINURAD))

Where CLTYP is typical value of clearance and k is a coefficient for verinurad dose (1/mg).

Parameter estimates from the final model can be found in the Table 4. Model diagnostics (Fig. 2 and Fig. 4) indicated that the pharmacokinetic data of oxypurinol is well described using the final model.

As expected, verinurad had clear impact on oxypurinol exposure. The coefficient for the clearance effect of the verinurad dose was estimated at 0.042, which means that verinurad increased oxypurinol clearance and therefore decreased oxypurinol systemic exposure in a dose-dependent manner. According to the model, oxypurinol exposure was decreased by approximately 30% when administered together with 12 mg verinurad. The impact of verinurad dose on oxypurinol exposure is demonstrated in Fig. 3.

# References

1. Wright DF, Duffull SB, Merriman TR, Dalbeth N, Barclay ML, Stamp LK (2016) Predicting allopurinol response in patients with gout. Br J Clin Pharmacol 81 (2):277-289. doi:10.1111/bcp.12799

2. Kankam M, Hall J, Gillen M, Yang X, Shen Z, Lee C, Liu S, Miner JN, Walker S, Clauson V, Wilson D, Nguyen M (2018) Pharmacokinetics, Pharmacodynamics, and Tolerability of Concomitant Multiple Dose Administration of Verinurad (RDEA3170) and Allopurinol in Adult Male Subjects With Gout. J Clin Pharmacol 58 (9):1214-1222. doi:10.1002/jcph.1119

3. Iwanaga T, Kobayashi D, Hirayama M, Maeda T, Tamai I (2005) Involvement of uric acid transporter in increased renal clearance of the xanthine oxidase inhibitor oxypurinol induced by a uricosuric agent, benzbromarone. Drug Metab Dispos 33 (12):1791-1795. doi:10.1124/dmd.105.006056

Table 1. Summary of clinical studies included in allopurinol popPK modeling.

| Study | Description | Population | Treatments |
| --- | --- | --- | --- |
| RDEA3170-107 | Verinurad and allopurinol combination study in gout subjects | Symptomatic hyperuricemic | • 300 mg allopurinol  • 10 mg verinurad + 300 mg allopurinol |
| RDEA3170-206 | Phase 2a verinurad and allopurinol combination study in gout subjects | Symptomatic hyperuricemic | • 300 mg allopurinol od  • 300 mg allopurinol bid  • 600 mg allopurinol  • 2.5 mg verinurad + 300 mg allopurinol  • 5 mg verinurad + 300 mg allopurinol  • 7.5 mg verinurad + 300 mg allopurinol  • 10 mg verinurad + 300 mg allopurinol  • 15 mg verinurad + 300 mg allopurinol  • 20 mg verinurad + 300 mg allopurinol |
| D5495C00006 | Multiple dose study in Asians/Chinese | Healthy volunteers | • 12 mg verinurad + 300 mg allopurinol  • 24 mg verinurad + 300 mg allopurinol |

bid, twice daily; od, once daily; popPK, population pharmacokinetic.

Table 2. Schedules for PK, sUA and uUA sampling for the studies included in the analysis

| Study | PK sampling schedule | sUA sampling schedule | uUA sampling schedule |
| --- | --- | --- | --- |
| RDEA3170-107 | - Days 7, 14, and 21: predose and 1, 2, 3, 4, 5, 6, 12, 22, and 24 h postdose | - Day –1: –24, –23, –22, –21, –20, –19, –18, –12, and –2 h prior to dosing on Day 1 and at predose - Days 7, 14, and 21: predose and 1, 2, 3, 4, 5, 6, 12, 22, and 24 h postdose | - Day –1: –24 to –23, –23 to –22, –22 to –21, –21 to –20, –20 to –19, –19 to –18, –18 to –16, –16 to –14, –14 to –12, –12 to  –2, and –2 to 0 h predose - Days 7, 8, 14, and 21: 0 to 1, 1 to 2, 2 to 3, 3 to 4, 4 to 5, 5 to 6, 6 to 8, 8 to 10, 10 to 12, 12 to 22, and 22 to 24 h postdose |
| RDEA3170-206 | - Days 7, 14, 21, 28, and 35: predose^*^ and 1, 2, 3, 4, 5, 6, 8, 10, 12, 22, and 24 h postdose | - Day –1: –24, –23, –22, –21, –20, –19, –18, –16, –14, –12, and –2 h prior to dosing on Day 1, and at predose^*^ - Days 7, 14, 21, 28, and 35: predose and 1, 2, 3, 4, 5, 6, 8, 10, 12, 22, and 24 h postdose | -^†^ |
| D5495C00006 | - Day 1 and Day 9: predose, 0.5, 1, 1.5, 2, 3, 4, 5, 6, 8, 10, 12 and 24 h postdose - Day 2: 36 h postdose - Days 3 to 8: predose | - Day –1: –24, –21, –18, and –12 h predose - Days 1 and 7: predose, 3, 6, 12, and 24 h postdose | - Day –1: baseline collection of urine: –24 to –22, –22 to –20, –20 to –18, –18 to –16, –16 to –14, –14 to –12 and –12 to 0 h predose - Days 1 and 7: 0 to 2, 2 to 4, 4 to 6, 6 to 8, 8 to 10, 10 to 12, and 12 to 24 h postdose |

*within 30 minutes prior to dosing. ^†^Urinary samples were not included in the analysis for this study due to an error with urine collection during the study. PK: pharmacokinetic, sUA: serum uric acid, uUA: urinary uric acid.

Table 3. Summary of baseline characteristics of patients included in the analysis

| **Study** | **n** | **Age (years) median  (min, max)** | **Body weight (kg) median (min, max)** | **eGFR (mL/min/1.73 m^2^) median (min, max)** | **Sex (Males) n (%)** | **Race: Caucasian n (%)** | **Race: Black n (%)** | **Race: Asian n (%)** | **Race: Other n (%)** |
| --- | --- | --- | --- | --- | --- | --- | --- | --- | --- |
| D5495C00006 | 18 | 38 (27, 48) | 71.9 (58.75, 90.75) | 101.9 (70.4, 117.8) | 17 (94.4) | 0 (0) | 0 (0) | 18 (100) | 0 (0) |
| RDEA3170-107 | 12 | 51.5 (29, 69) | 99.7 (80.9, 126.3) | 84.3 (54.1, 102.9) | 12 (100) | 7 (58.3) | 4 (33.3) | 0 (0) | 1 (8.3) |
| RDEA3170-206 | 40 | 49 (28, 74) | 92.9 (63.1, 147.6) | 89.5 (55.1, 125.6) | 39 (97.5) | 30 (75) | 6 (15) | 4 (10) | 0 (0) |
| All subjects | 70 | 46 (27, 74) | 89.7 (58.75, 147.6) | 93 (54.1, 125.6) | 68 (97.1) | 37 (52.9) | 10 (14.3) | 22 (31.4) | 1 (1.4) |

eGFR, estimated glomerular filtration rate.

Table 4. Parameter estimates of the final oxypurinol popPK model.

| **Parameter** | **Estimate** | **RSE, %** | **IIV (CV%)** | **RSE, %** |
| --- | --- | --- | --- | --- |
| Central clearance (L/h) | 1.373 | 4.31 | 24.11 | 9.89 |
| Central volume of distribution (L) | 45.69 | 4.78 | 30.88 | 37.4 |
| Inter-compartmental clearance (L/h) | 2.488 | 7.32 |  |  |
| Peripheral volume of distribution (L) | 23.80 | 11.7 |  |  |
| Absorption rate constant (1/h) | 0.3343 | 7.12 | 36.64 | 23.2 |
| Coefficient for verinurad dose (1/mg) | 0.04200 | 20.4 |  |  |
| BW ~ central volume of distribution | 0.7926 | 32.2 |  |  |
| eGFR ~ central clearance | 0.3821 | 33.0 |  |  |
| Proportional error | 0.1941 | 10.9 |  |  |

BW, body weight; eGFR, estimated glomerular filtration rate; popPK, population pharmacokinetic; IIV, interindividual variability; RSE, relative standard error.

Fig 1. Observed pharmacokinetic data from allopurinol studies.

**
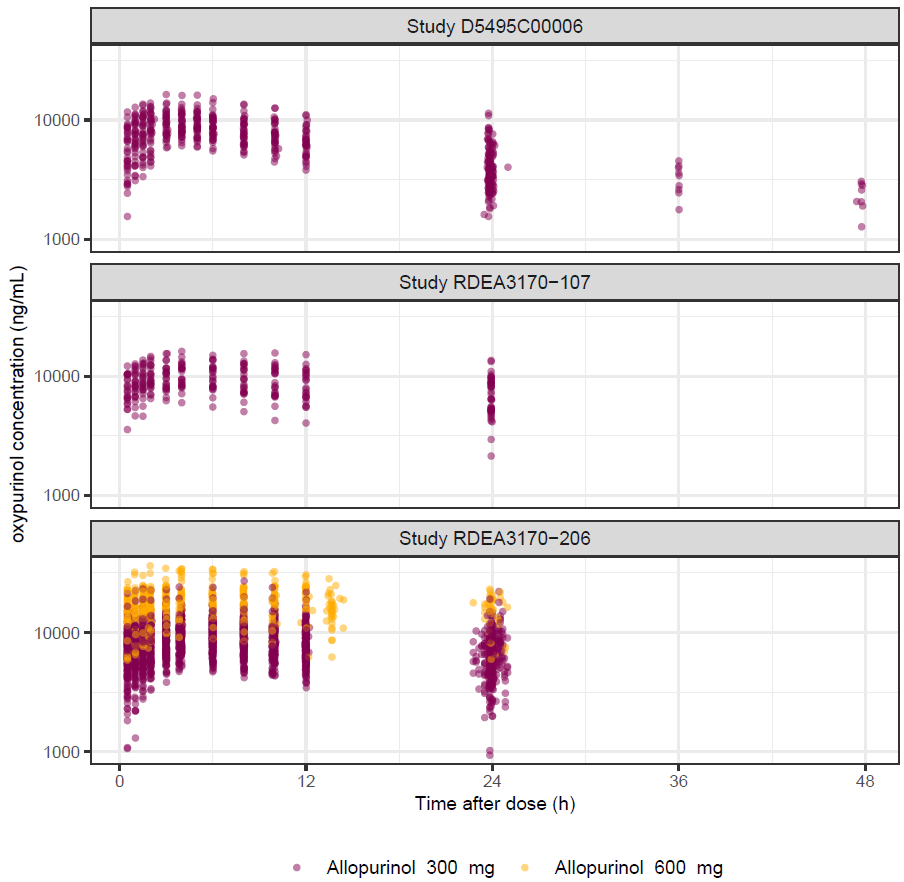
**

Fig. 2. Goodness-of-fit plots for the final oxypurinol popPK model. Red line is the line of unity (top-left and middle, and bottom-right), or reference line of zero (bottom-left and middle), or normal distribution with mean of zero and estimated residual variance (top-right). Blue line is the non-parametric smoother (left and middle) or smoothed density line of the residuals (top-right).


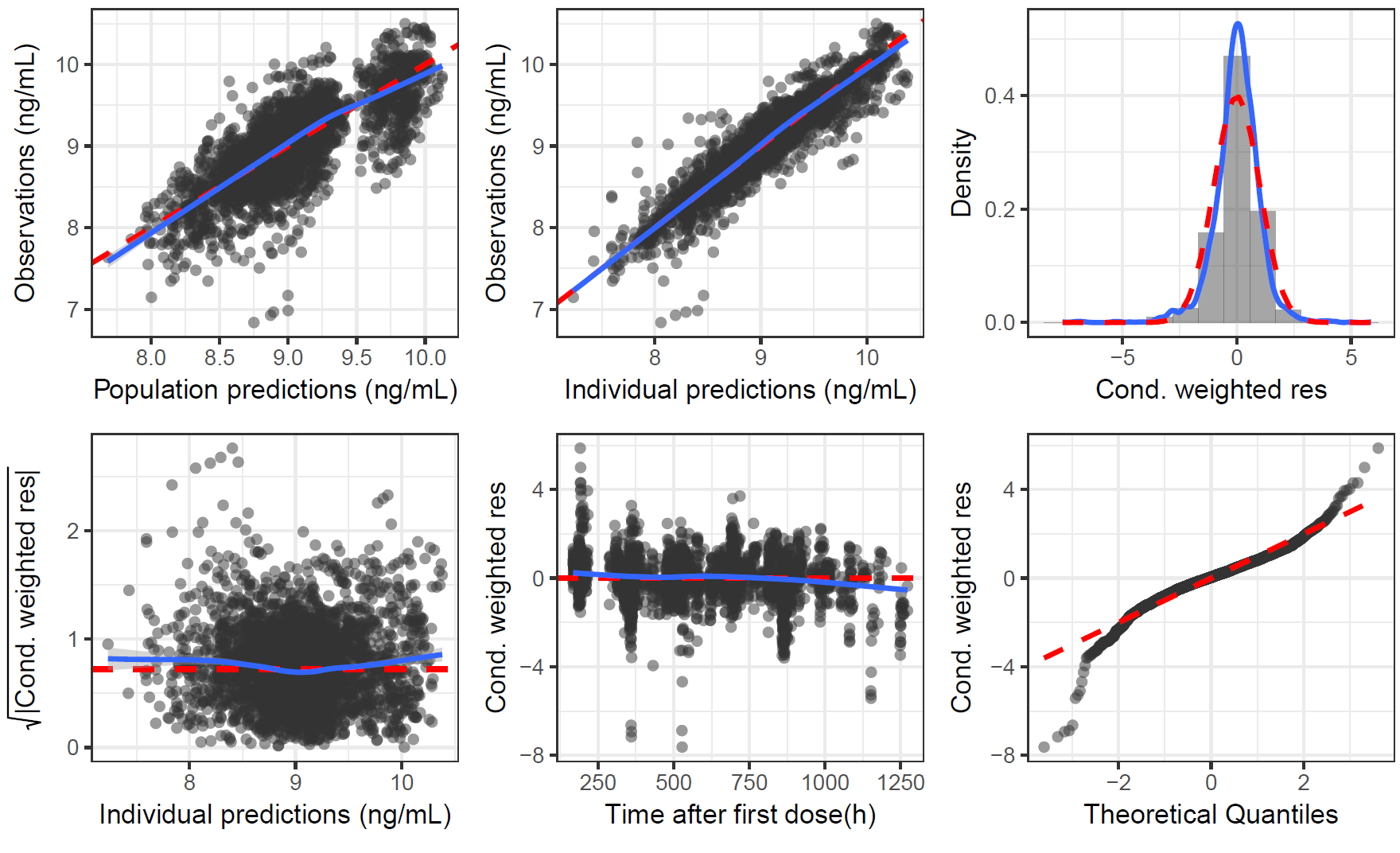


popPK, population pharmacokinetic.

Fig. 3. The impact of verinurad dose on oxypurinol exposure


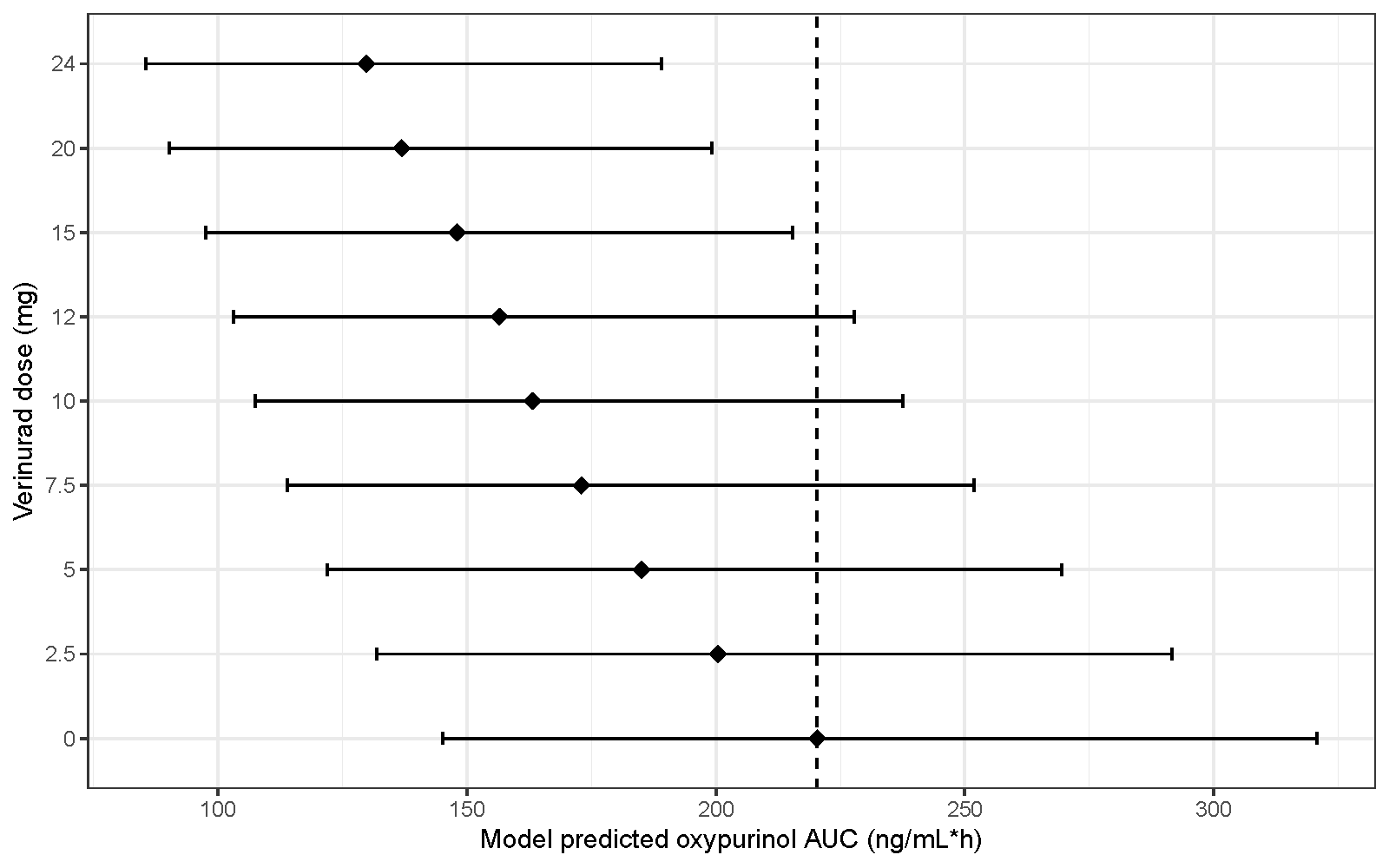


AUC, area under the curve.

Fig. 4. Prediction corrected visual predictive check plot for the final oxypurinol popPK model


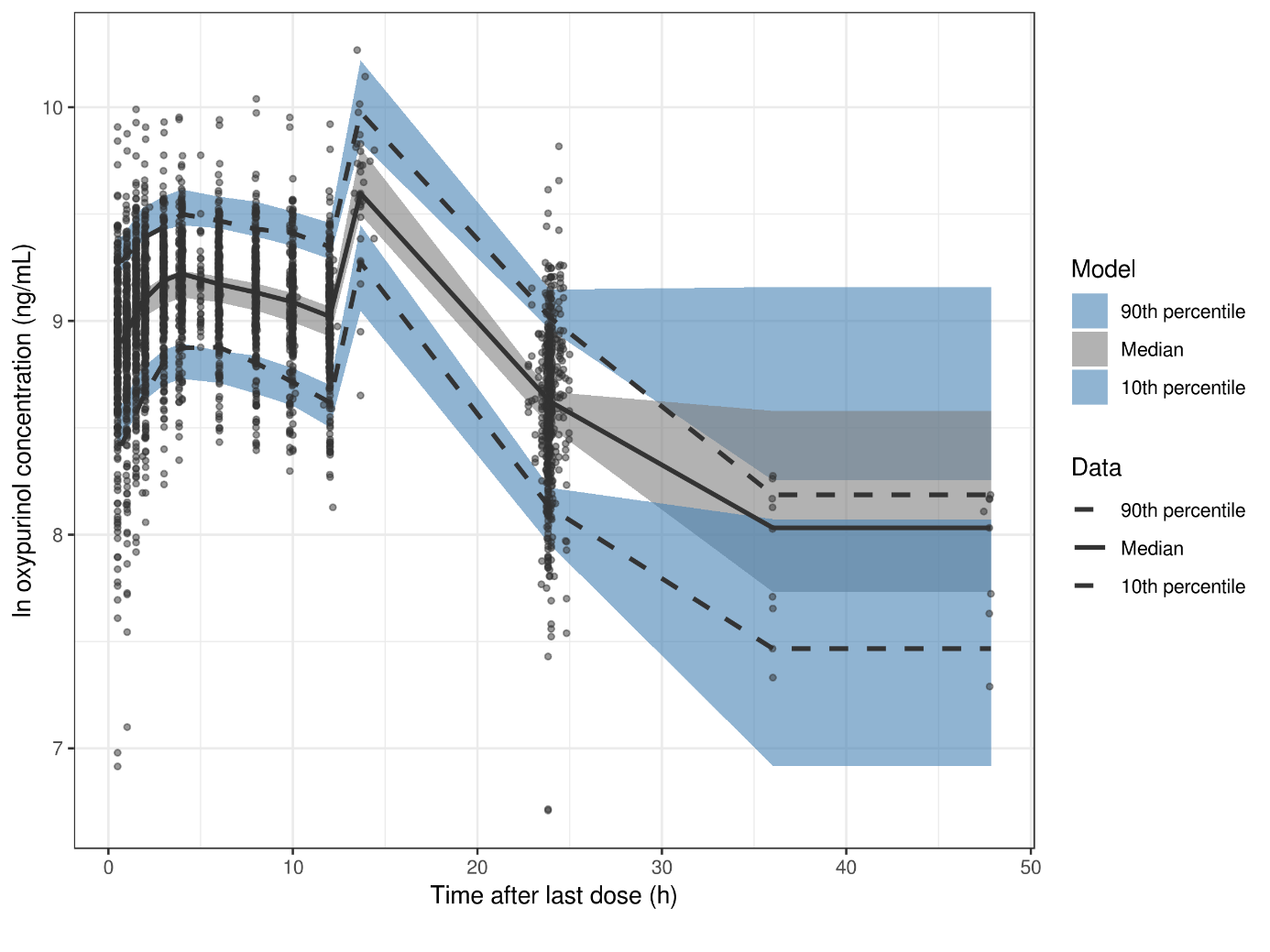


popPK, population pharmacokinetic.
