# Supplementary material for: A semi-mechanistic exposure–response model to assess the effects of verinurad, a potent URAT1 inhibitor, on serum and urine uric acid in patients with hyperuricemia-associated diseases
Source: J Pharmacokinet Pharmacodyn. 2021 Mar 17;48(4):525–41. doi: 10.1007/s10928-021-09747-y (PMC8225519; doi:10.1007/s10928-021-09747-y)
Supplement: Supplementary file 4 — Supplementary file4 (DOCX 1142 kb) [file 10928_2021_9747_MOESM4_ESM.docx]

A semi-mechanistic exposure-response model to assess the effects of verinurad, a potent URAT1 inhibitor, on serum and urine uric acid in patients with hyperuricemia-associated diseases

**Jacob Leander^1^, Mikael Sunnåker^1^, Dinko Rekić^1^, Sergey Aksenov^2^, Ulf G. Eriksson^1^, Susanne Johansson^1^, Joanna Parkinson^1^**

*^1^Clinical Pharmacology and Quantitative Pharmacology, Clinical Pharmacology and Safety Sciences, R&D, AstraZeneca, Gothenburg, Sweden*

*^2^Clinical Pharmacology and Quantitative Pharmacology, Clinical Pharmacology and Safety Sciences, R&D, AstraZeneca, Waltham*

**Corresponding author**

Joanna Parkinson

email: [Joanna.parkinson@astrazeneca.com](mailto:Joanna.parkinson@astrazeneca.com)

# Supplementary material 4. Uric acid model goodness-of-fits and model qualification

In Fig. 1, a panel of standard goodness-of-fit plots for the serum uric acid observations are shown. In Fig. 2 (daily urine collections) and Fig. 3 (including nightly urine collection), the corresponding goodness-of-fit plots for the urine observations are shown. Overall, there is a good agreement between model prediction and observations. There is a slight overprediction for the longest urine interval (overnight) collection. A potential reason for this is that diurnal variation might lead to an excretion rate that decreases during the night, something that is not included in the model, as the model assumes a constant excretion rate over any urine collection period.

In Fig. 4, Fig. 5, Fig. 6, and Fig. 7, the population predictions (PRED) versus observations (DV) and individual predictions (IPRED) versus observations (DV) are shown stratified by study. Overall, there is a good agreement for all studies.

The visual predictive check (VPC) is a valuable tool for assessing the model’s ability to capture the variability in the data. The integrated uric acid model is built on a large set of clinical studies with several combinations of doses and durations. To show the model’s ability to describe the observed data, we present a collection of visual predictive checks meant to illustrate a wide range of scenarios.

In Fig. 8, prediction-corrected VPC for uric acid observations in a single dose (4.5 mg, 6 mg, or 12 mg verinurad ER8) in healthy volunteers is shown (study RDEA3170-112). Overall, good agreement with observations is seen, however there is some overprediction for urinary uric acid.

In Fig. 9, the prediction-corrected VPC for serum uric acid observations in a phase IIa allopurinol combination study (RDEA3170-206) in symptomatic hyperuricemic patients is shown. Doses included 300 mg allopurinol with and without 2.5 mg, 5 mg, 7.5 mg, 10 mg, 15 mg, 20 mg verinurad MR4, and 600 mg allopurinol. A good agreement with observations is seen.

In Fig. 10, the prediction-corrected VPC for the febuxostat combination study in asymptomatic hyperuricemics (study D5495C00001, 9 mg verinurad ER8 in combination with 80 mg febuxostat) is shown. Overall, good agreement with observations is seen, however there is some overprediction for urinary uric acid.

In Fig. 11 and Fig. 12, the prediction-corrected VPCs for serum and urine acid observations in the renal impairment study RDEA3170-108 (single dose verinurad 15 mg MR4) are shown. For the more severe renally impaired patients, it is evident that the model seems to overpredict serum and urine uric acid levels, which is also indicated in the PRED-DV plot in Figure 4 for the same study. The reason for this might potentially be due to higher fractional excretion in renally impaired patients, which affects serum uric acid level and the effect of the treatment. The reason for a higher fractional excretion of uric acid in this patient population is not fully understood but it might be attributed to the effect of kidney disease on activity of URAT1 or other uric acid transporters.

To validate the model’s ability to describe data not used to build the pharmacodynamic model, a prediction-corrected VPC for the study D5495C00006 is provided. This was a study with either 12 mg or 24 mg verinurad combined with 300 mg allopurinol once daily in Asian subjects. The validation VPC is seen in Fig. 13, which shows a good agreement with observations and model prediction, although the model predicts slightly higher steady-state sUA values than observed.

Fig. 1. Goodness-of-fits for the for serum uric acid measurements. The red line is the line of unity (top-left and middle, and bottom-right), or reference line of zero (bottom-left and middle), or normal distribution with mean of zero and estimated residual variance (top-right). The blue line is the non-parametric smoother (left and middle) or smoothed density line of the residuals (top-right)


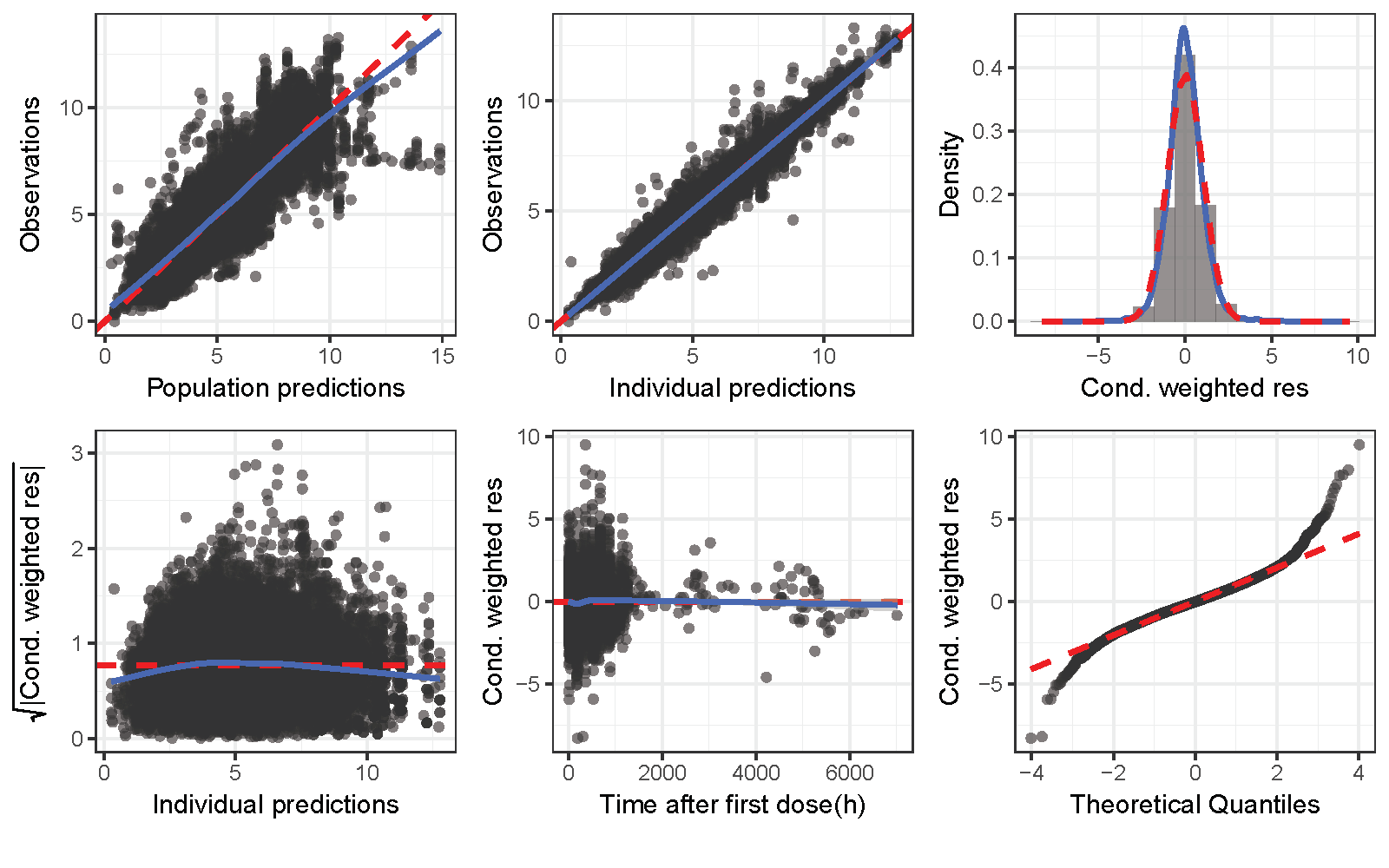


Observations and predictions correspond to serum uric acid, mg/dL

Fig. 2. Goodness-of-fits for the for urine uric acid measurements, daily urine collections. The red line is the line of unity (top-left and middle, and bottom-right), or reference line of zero (bottom-left and middle), or normal distribution with mean of zero and estimated residual variance (top-right). The blue line is the non-parametric smoother (left and middle) or smoothed density line of the residuals (top-right)


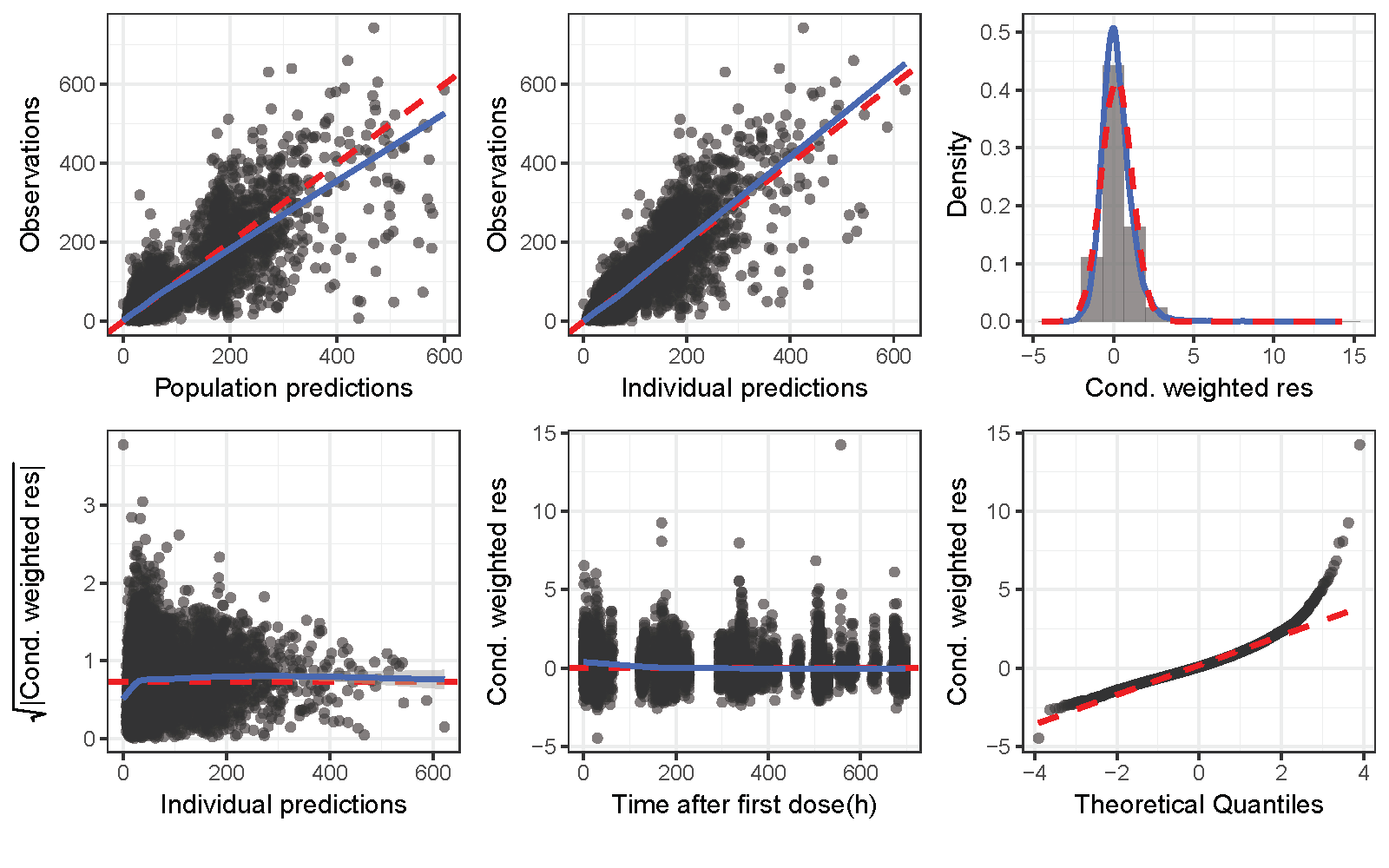


Observations and predictions correspond to urinary uric acid, mg

Fig. 3. Goodness-of-fits for the for urine uric acid measurements, including nightly urine collections. The red line is the line of unity (top-left and middle, and bottom-right), or reference line of zero (bottom-left and middle), or normal distribution with mean of zero and estimated residual variance (top-right). The blue line is the non-parametric smoother (left and middle) or smoothed density line of the residuals (top-right)


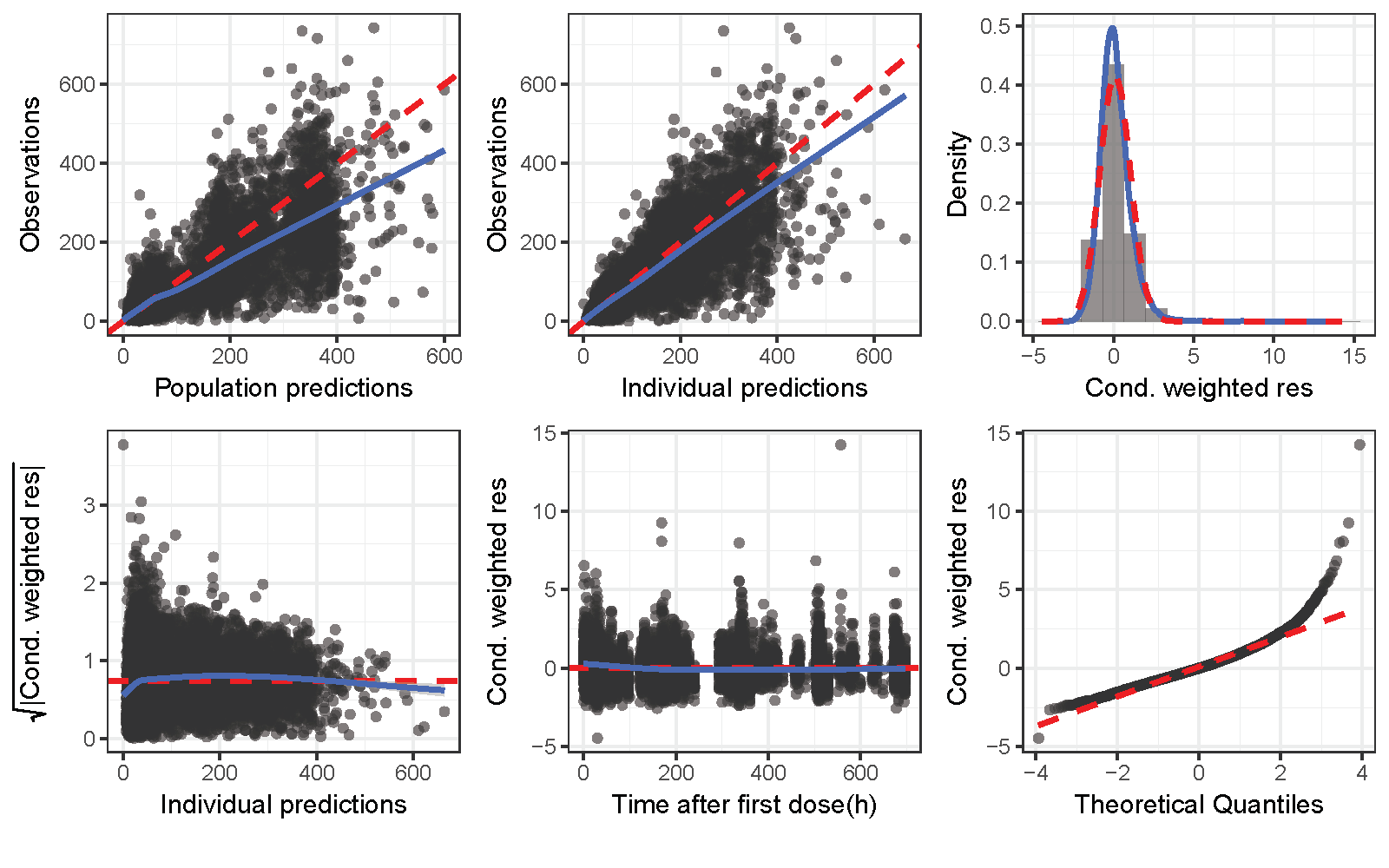


Observations and predictions correspond to urinary uric acid, mg

Fig. 4. Goodness-of-fits for serum uric acid measurements, showing population predictions (PRED) versus observations (DV) stratified on study number


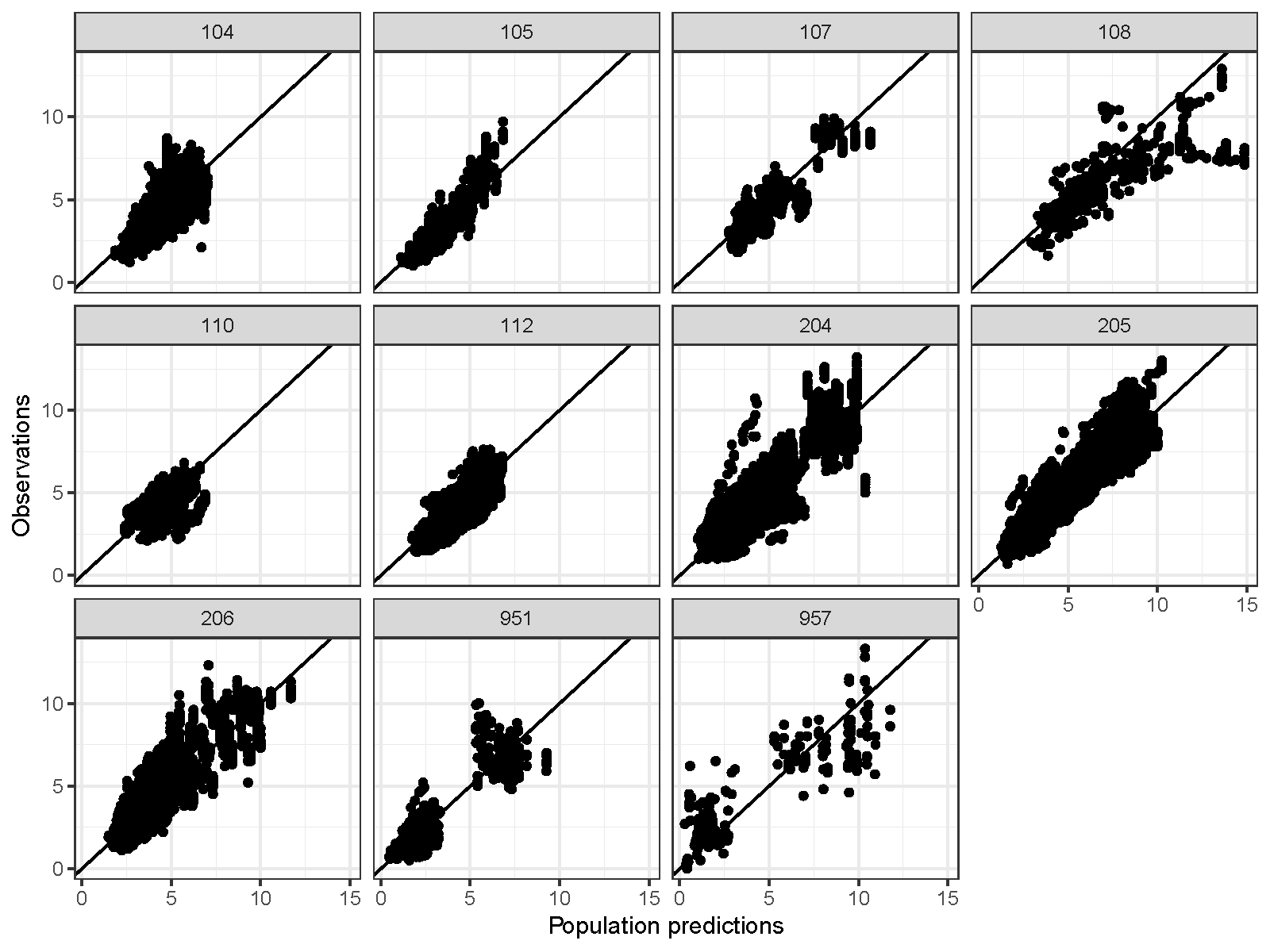


Observations and predictions correspond to serum uric acid, mg/dL

Fig. 5. Goodness-of-fits for serum uric acid measurements, showing individual predictions (IPRED) versus observations (DV) stratified on study number


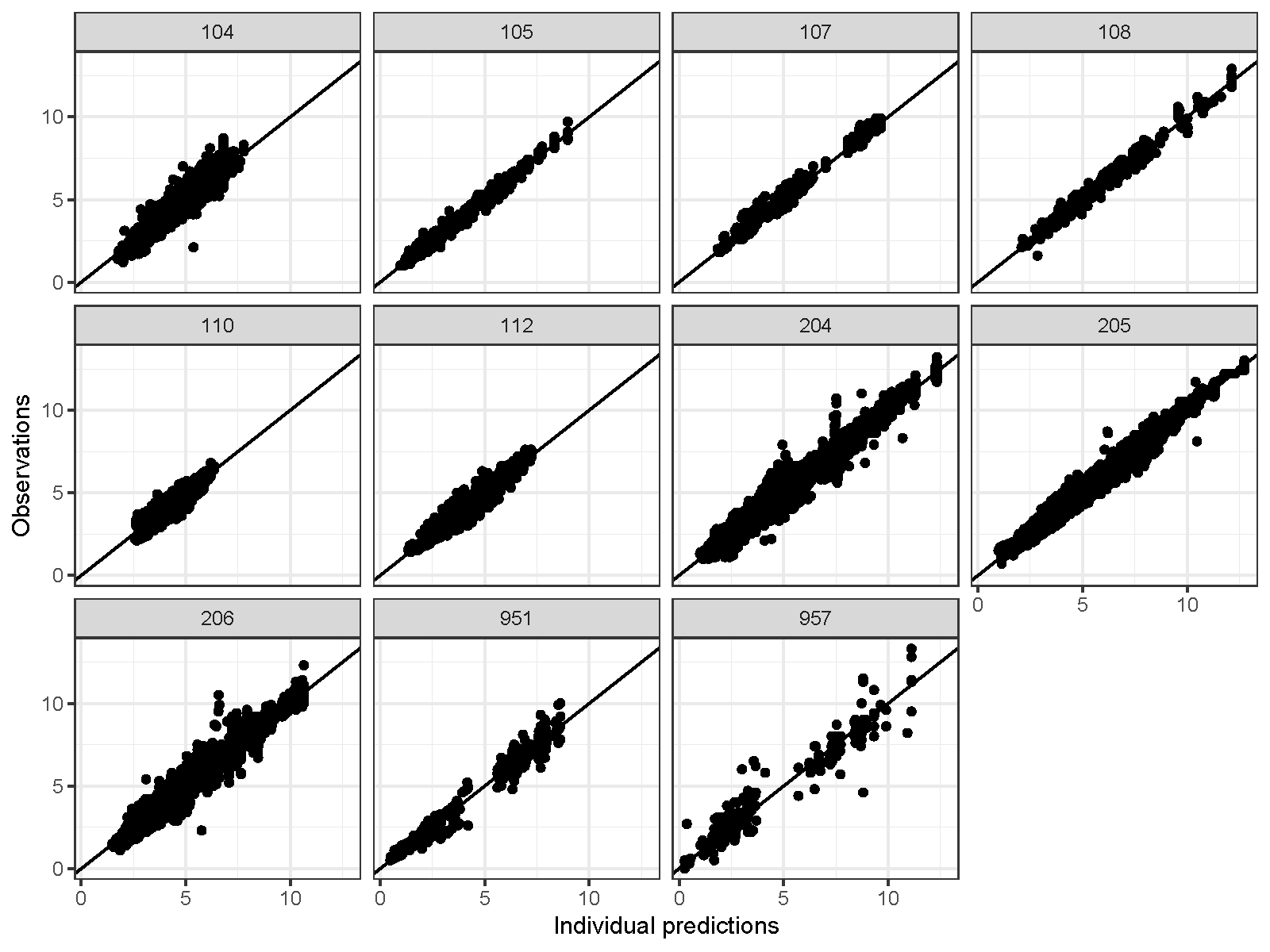


Observations and predictions correspond to serum uric acid, mg/dL

Fig. 6. Goodness-of-fits for urine uric acid measurements, showing population predictions (PRED) versus observations (DV) stratified on study number


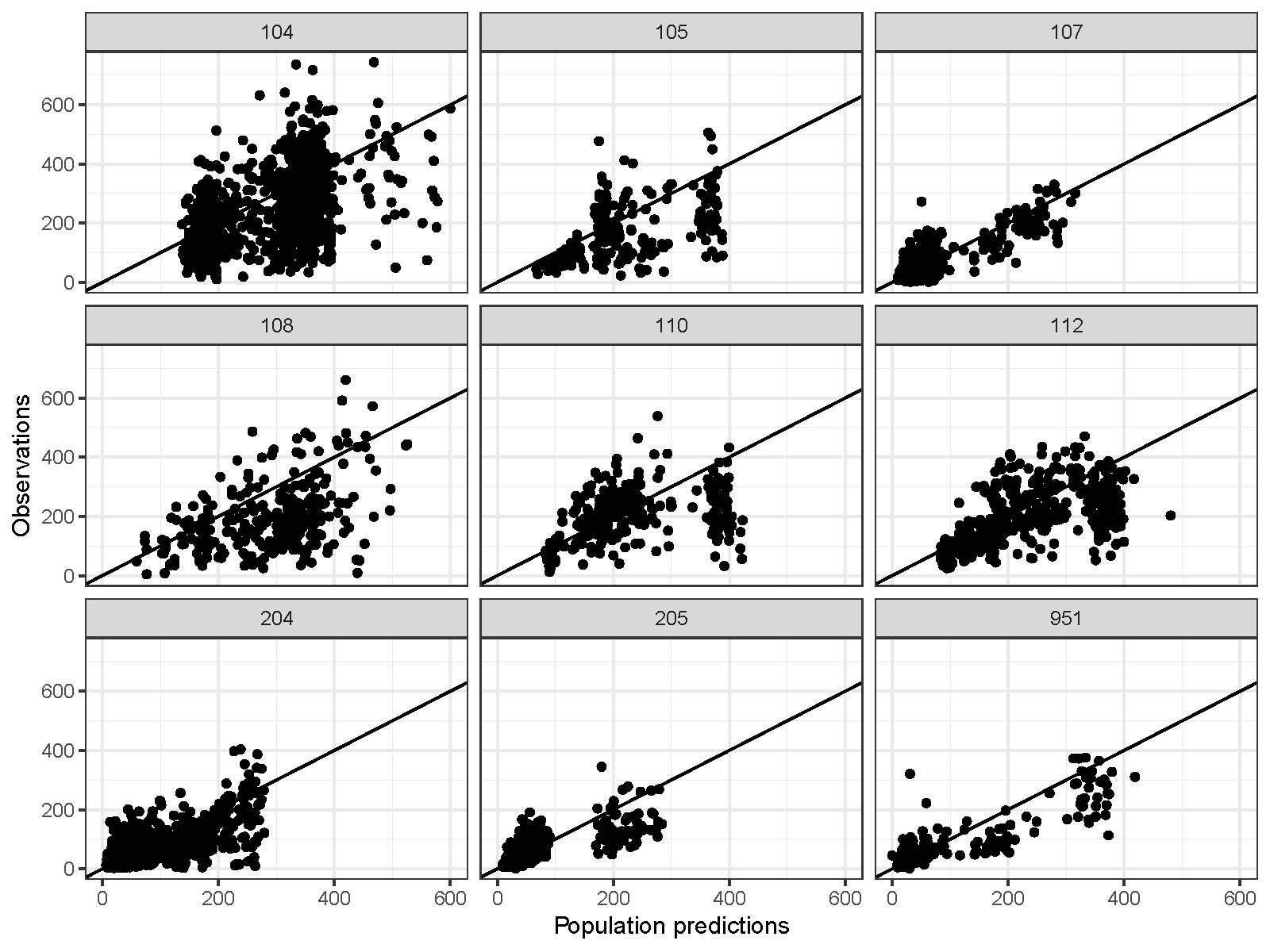


Observations and predictions correspond to urinary uric acid, mg

Fig. 7. Goodness-of-fits for urine uric acid measurements, showing individual predictions (IPRED) versus observations (DV) stratified on study number


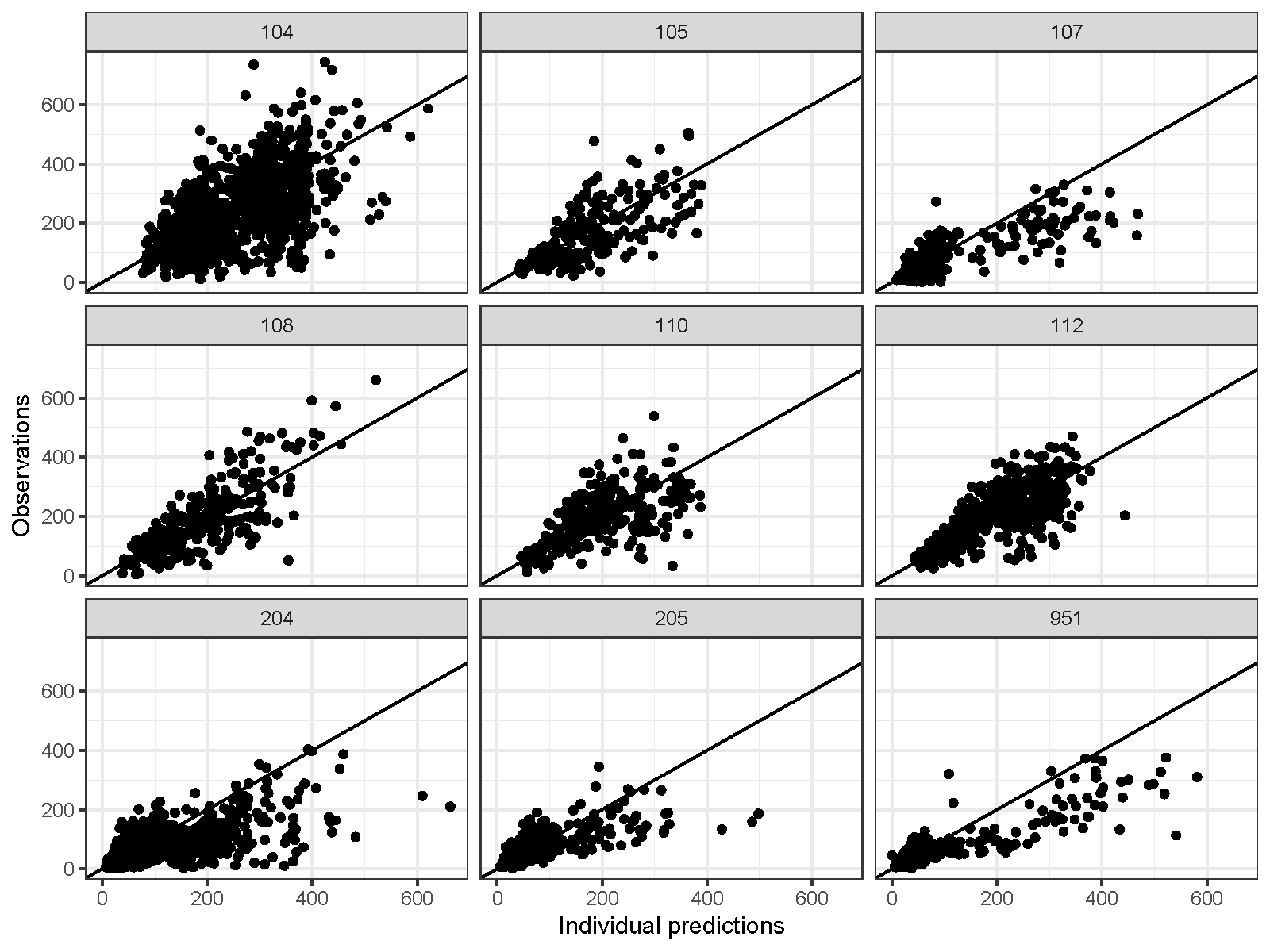


Observations and predictions correspond to urinary uric acid, mg

Fig. 8. Prediction-corrected VPC for study RDEA3170-112 (single dose 4.5 mg, 6 mg, 12 mg verinurad ER8) for uric acid observations (upper panel serum, lower panel urine). The solid and dashed lines are the median and the 10th and 90th percentiles of the observations. The shaded areas are the 95% confidence intervals of the median and the 10th and 90th percentiles predicted by the model. The symbols are observed data


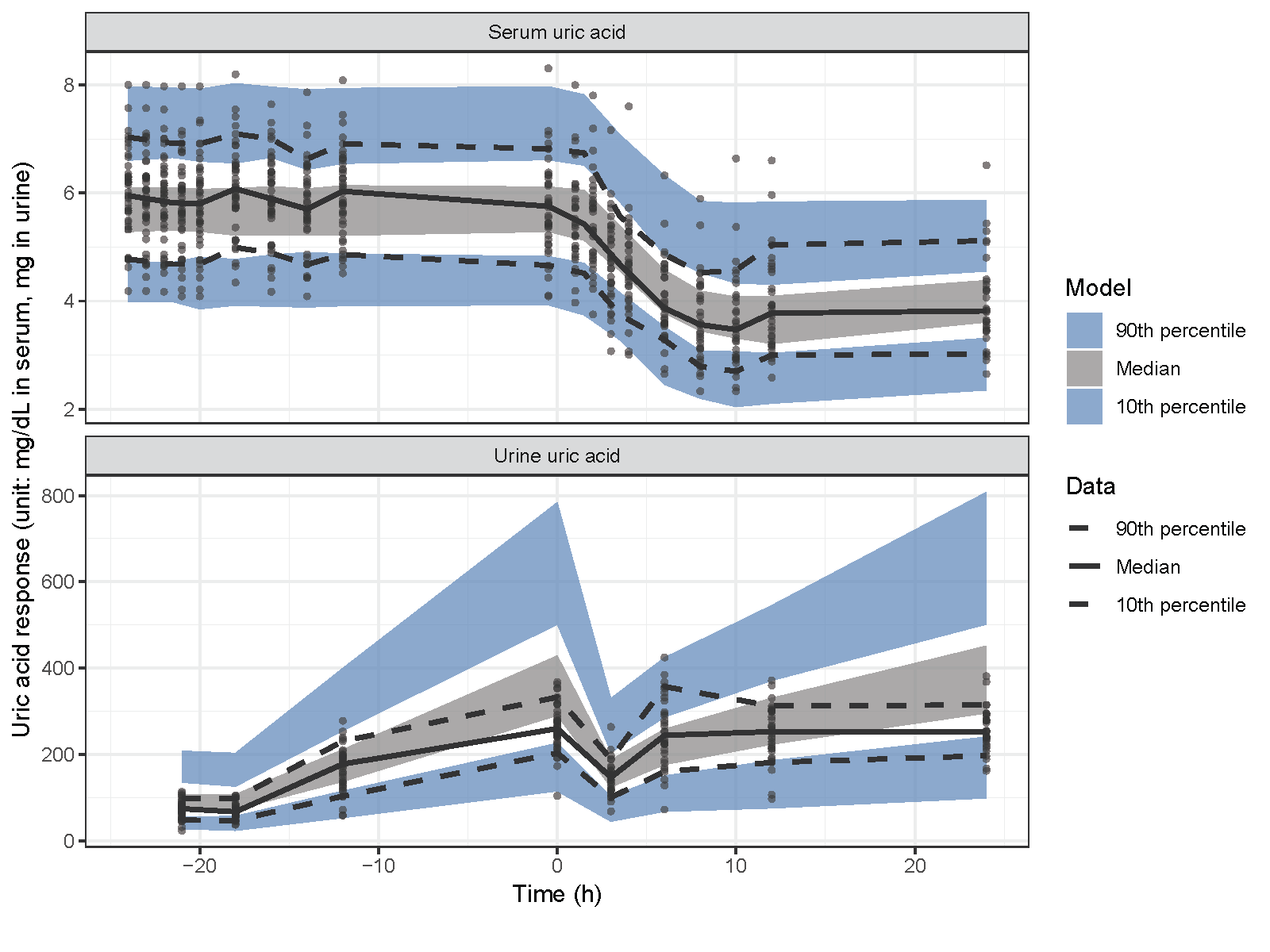


ER8, extended release 8; VPC, visual predictive check.

Fig. 9. Prediction-corrected VPC for the allopurinol combination study RDEA3170-206 (300 mg allopurinol with and without 2.5 mg, 5 mg, 7.5 mg, 10 mg, 15 mg, 20 mg verinurad MR4, and 600 mg allopurinol) for serum uric acid observations. The solid and dashed lines are the median and the 10th and 90th percentiles of the observations. The shaded areas are the 95% confidence intervals of the median and the 10th and 90th percentiles predicted by the model. The symbols are observed data


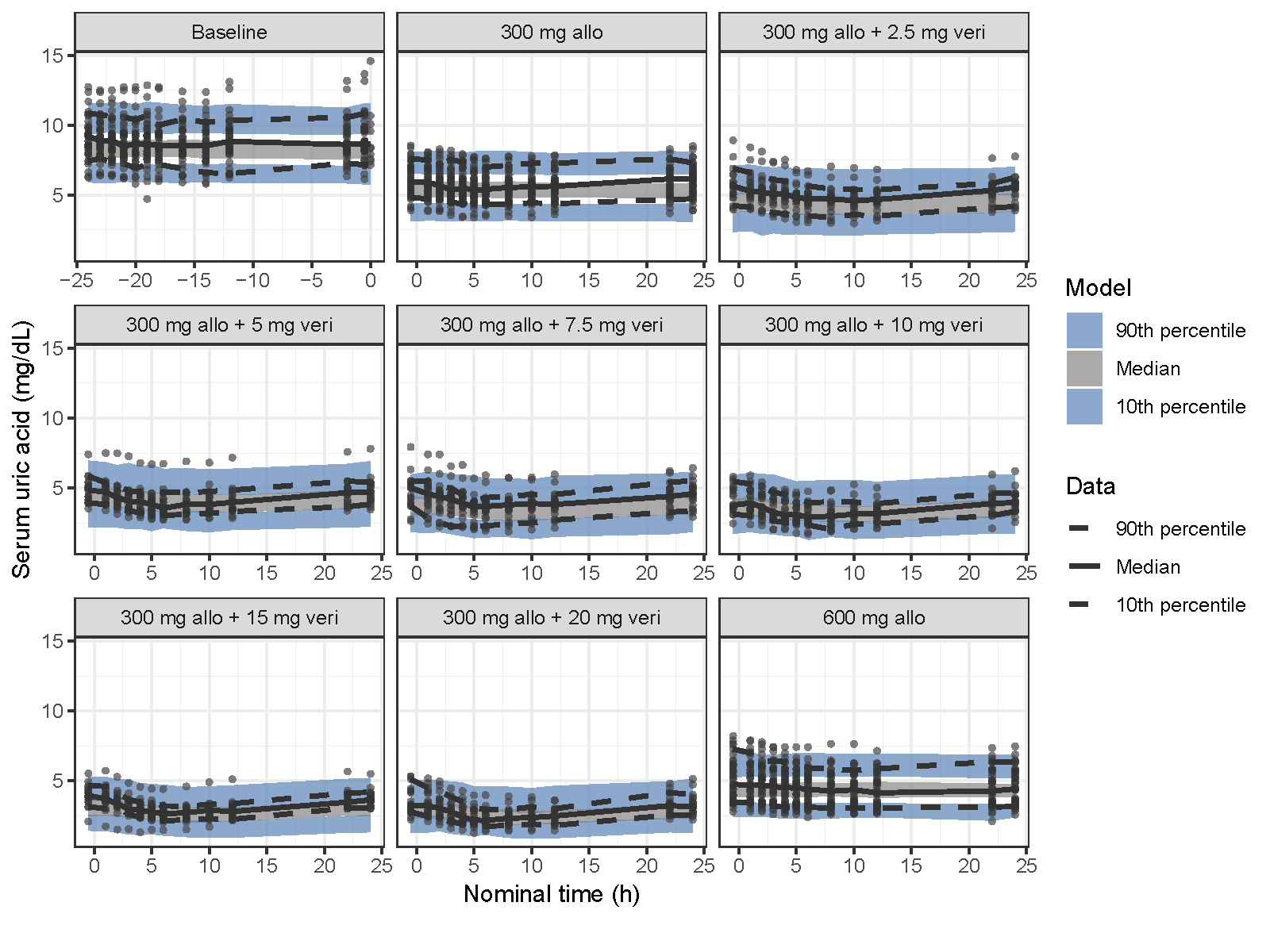


allo, allopurinol; MR4, modified release; veri, verinurad; VPC, visual predictive check.

Fig. 10. Prediction-corrected VPC for the febuxostat combination study D5495C00001 (9 mg verinurad ER8 + 80 mg febuxostat) in hyperuricemic subjects for uric acid observations (upper panels serum, lower panels urine). The solid and dashed lines are the median and the 10th and 90th percentiles of the observations. The shaded areas are the 95% confidence intervals of the median and the 10th and 90th percentiles predicted by the model. The symbols are observed data


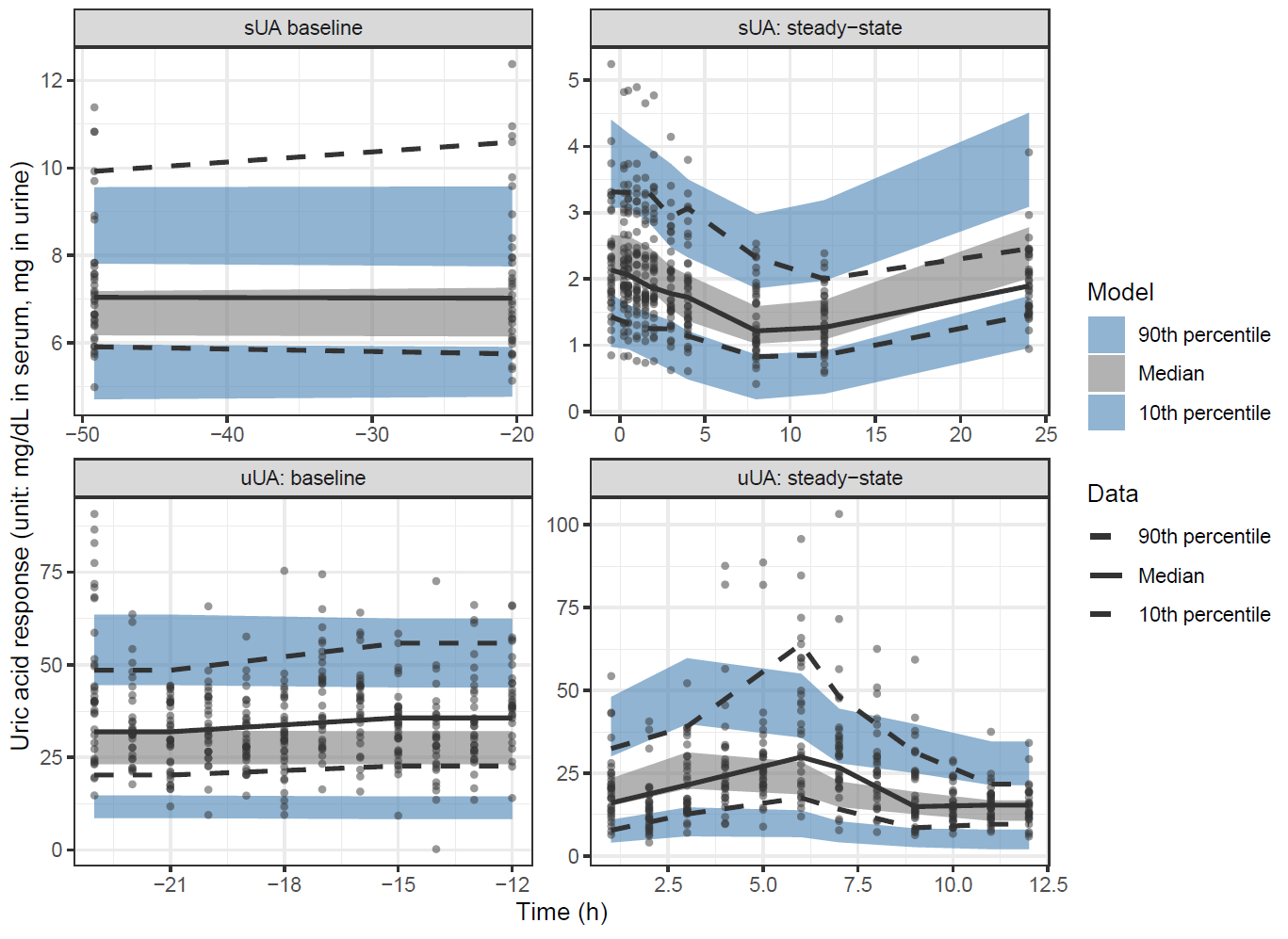


ER8, extended release; sUA, serum uric acid; uUA, urinary uric acid; VPC, visual predictive check.

Fig. 11. Prediction-corrected VPC for the serum uric acid observations for the renal impairment study RDEA3170-108 (15 mg verinurad MR4). The solid and dashed lines are the median and the 10th and 90th percentiles of the observations. The shaded areas are the 95% confidence intervals of the median and the 10th and 90th percentiles predicted by the model. The symbols are observed data


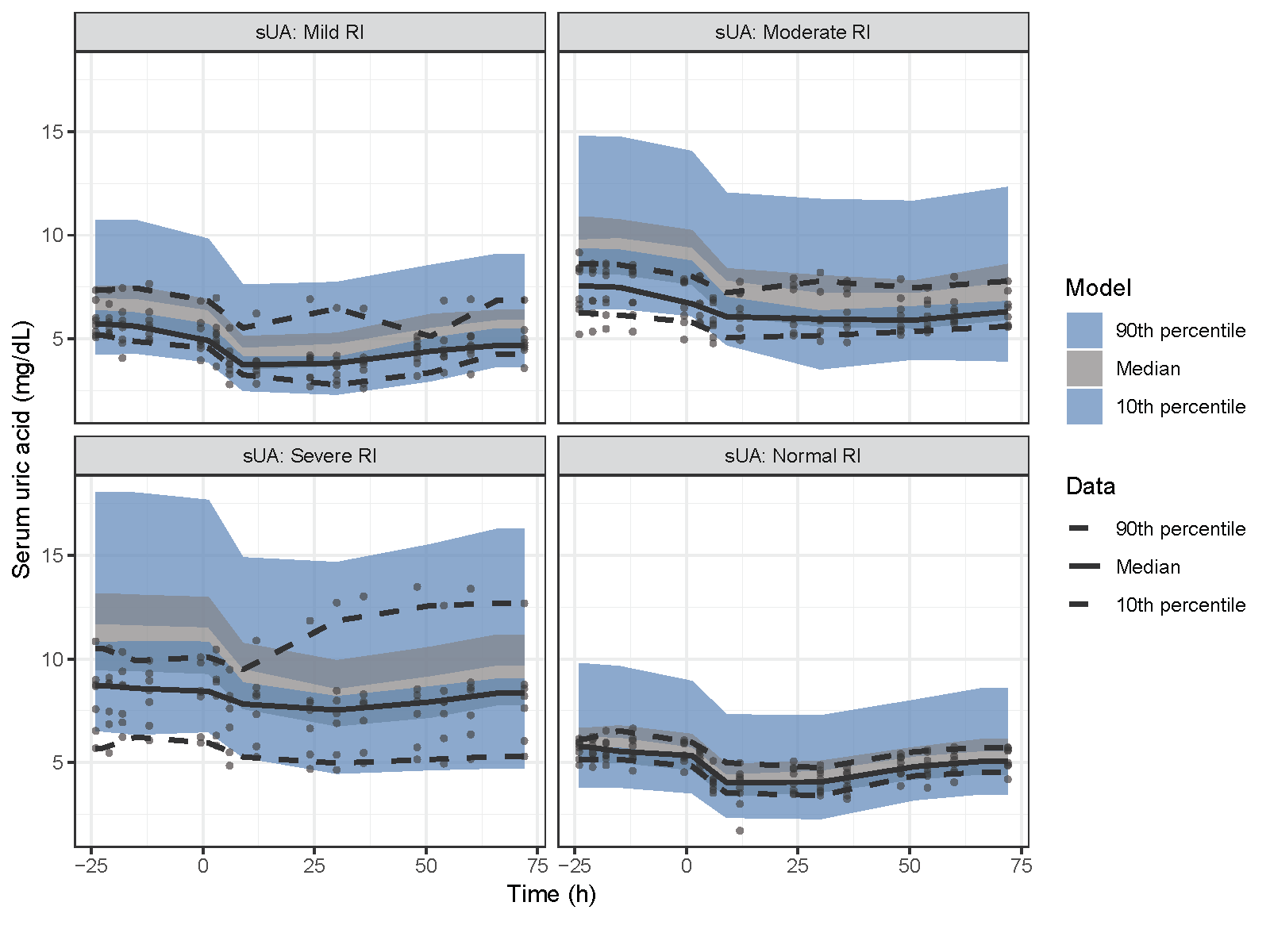


MR4, modified release; RI, renal impairment; sUA, serum uric acid; uUA, urinary uric acid; VPC, visual predictive check.

Fig. 12. Prediction-corrected VPC for the urine uric acid observations for the renal impairment study RDEA3170-108 (15 mg verinurad MR4). The solid and dashed lines are the median and the 10th and 90th percentiles of the observations. The shaded areas are the 95% confidence intervals of the median and the 10th and 90th percentiles predicted by the model. The symbols are observed data


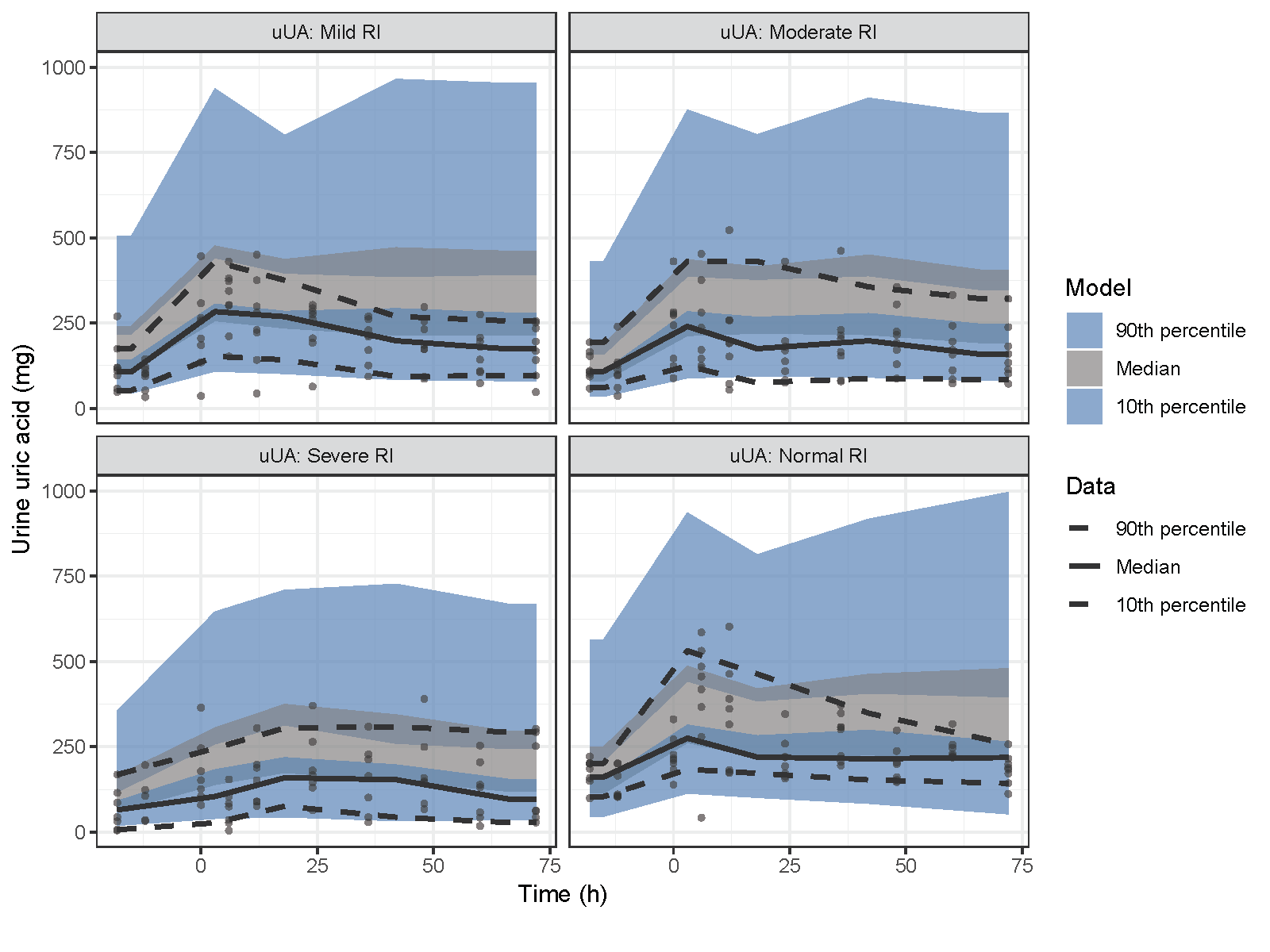


MR4, modified release; RI, renal impairment; uUA, urinary uric acid; VPC, visual predictive check.

Fig. 13. Prediction-corrected VPC for study D5495C00006 (12 mg or 24 mg verinurad ER8 in combination with 300 mg allopurinol) for uric acid observations (upper panels serum, lower panels urine). The solid and dashed lines are the median and the 10th and 90th percentiles of the observations. The shaded areas are the 95% confidence intervals of the median and the 10th and 90th percentiles predicted by the model. The symbols are observed data


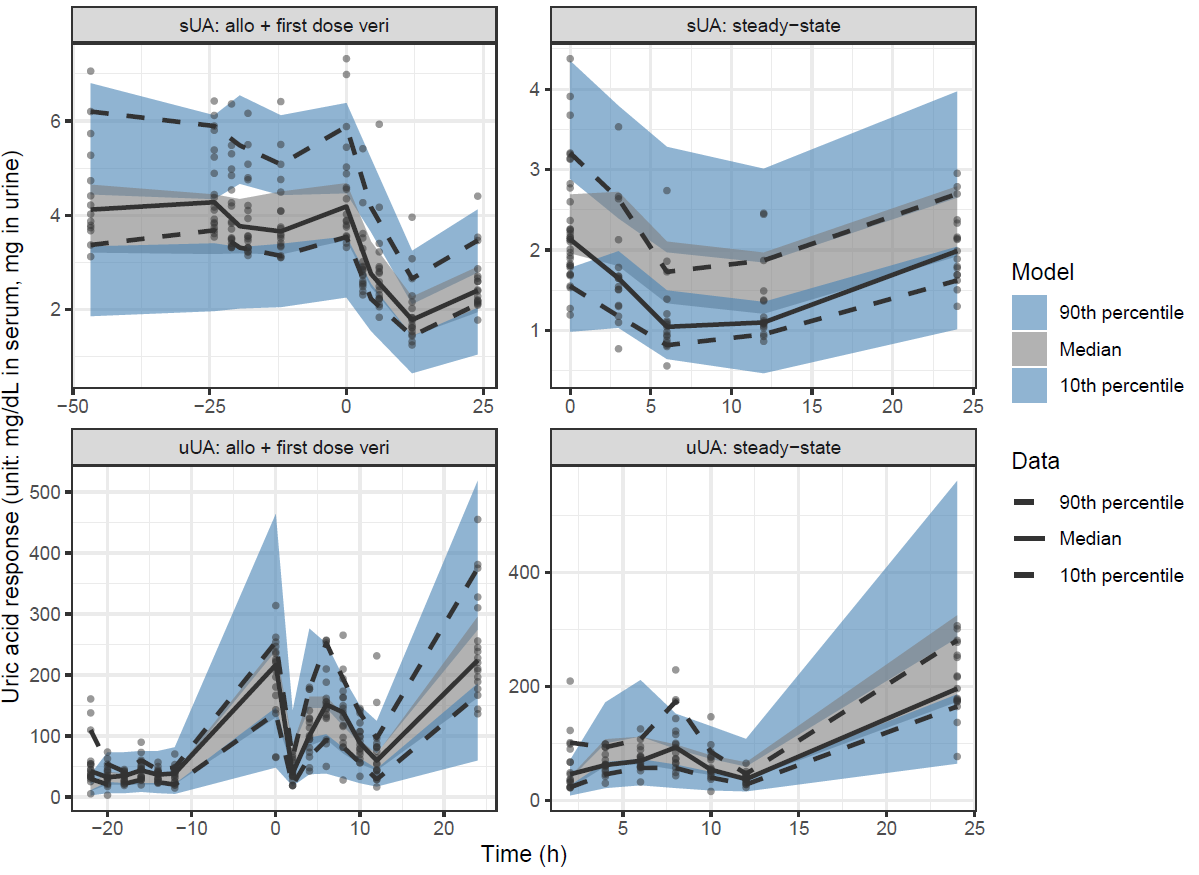


ER8, extended release; sUA, serum uric acid; uUA, urinary uric acid; VPC, visual predictive check; allo, allopurinol; veri, verinurad.
